# Effects of electrical biostimulation and silver ions on porcine fibroblast cells

**DOI:** 10.1371/journal.pone.0246847

**Published:** 2021-02-10

**Authors:** Yuanfeng Zhao, Thomas D. Bunch, S. Clay Isom

**Affiliations:** Department of Animal, Dairy and Veterinary Sciences, Utah State University, Logan, Utah, United States of America; University of Milano-Bicocca, ITALY

## Abstract

The medical applications of electrical biostimulation and silver ions have been evaluated in laboratory experiments and clinical studies for more than two decades. Their effects on preventing infection and promoting wound healing have been described. However, little is known about the role of electrical biostimulation and/or silver ion on changes in cellular transcriptome dynamics. To our knowledge, few studies have been conducted to investigate the potential of electrical biostimulation and silver ions in cell reprogramming. Besides, it is essential to assess any possible adverse effects or potential benefits of the silver ions on mammalian cells to address its safety concerns and to improve silver medical products. In this study, we investigated transcriptomic changes in porcine fibroblast cells in response to electrical biostimulation in the presence of silver ions. Exposed cells presented distinct morphological changes after treatment, which was mainly due to the exposure of silver ions rather than the electrical current itself. Gene expression analyses suggested that electrical biostimulation and silver ions did not increase the expression of pluripotency genes. Interestingly, a set of genes related to cellular metabolic processes were differentially expressed after cells were exposed to electrically generated silver ions for 21 hours. We found that 2.00 mg/L of electrically generated silver ion caused an increase of ATP generation and an increase of the total pool of NAD^+^ and NADH, while ROS production did not change. Aside from toxic effects, the results reported herein demonstrate the alternative effects of silver ions on mammalian cells, especially an oxidative phosphorylation burst. To our knowledge, this response of mammalian cells to silver ions has not been described previously. Although the function of this burst is not understood, it may lead to alterations in cellular activities such as metabolic remodeling and cell reprogramming, and/or serve an as-yet unknown function in neutralization or detoxification of the silver ions within the cells.

## Introduction

Bioelectric properties or signals play a key role in many biological processes and cell behaviors, such as proliferation, mitosis, apoptosis, migration, orientation, differentiation, and de-differentiation [1]. Electromagnetic biostimulation can alter bioelectric signals of cells, consequently affecting the behavior of cells. The electrical biostimulation is one of the simplest methods of electromagnetic biostimulation used to investigate how cells respond when exposed to electrical currents of different amplitudes and frequencies. This method has been applied to in vitro studies, animal experiments, and clinical trials for wound healing and bone regeneration [2–4]. For example, in the 1970s, R.O. Becker, an orthopedic surgeon and researcher, treated his patients with mild electrical stimulation in trials and reported positive effects on infection prevention, wound healing, and bone growth [5]. In another work following those trials, Becker demonstrated that the morphology and behavior of mammalian cells can be modified when they were subjected to a low-intensity direct current applied through a silver electrode. Treated cells clumped together into bits of pseudo-tissue resembling the differentiated young bone marrow cells [5], though the molecular characteristics of the modified cells were unverified. Given the prospects that electrical biostimulation can alter the epigenome [1], in this study, the primary goal was to determine if electrical stimulation alters gene expression in somatic cells. Yamanaka and Takahashi [6–8] demonstrated that by using a defined cocktail of transcription factors with a viral vector they could induce latent pluripotency into differentiated somatic cells, resulting in a transformation from fibroblast cells to a more primitive pluripotent state (iPS cells). The cocktail consisted of *KLF4*, *OCT4*, *SOX2*, and *c-MYC*, which have networks in cell fate determination and are definitely important to cellular reprogramming. The concept of cell transformation into a relatively undifferentiated state led us to consider the possibility that electrical field stimulation might also have the capability to directly induce de-differentiation or cellular reprogramming.

In the present work, a comprehensive, systematic evaluation of gene expression patterns in porcine fibroblasts exposed to a defined electrical stimulus was studied. The conducted experiments resembled Becker’s original experimental approaches as a starting point [5, 9], then modified to the optimal conditions based on the results. Specifically, Becker exposed fibroblast cells to silver electrodes and electrical current (360–380 nA) for 5 hours in his descriptive and clinical work. In order to maximize potential effects, a longer exposure time, 21 hours of stimulation was chosen as default in this study. The objectives of the present work were to determine in more depth the effects of electrical biostimulation on fibroblast cells. More specifically, we wanted to study the relationship between morphological changes and physiological changes (i.e. gene expression); to test the effects of the intensity of the current (and/or the concentration of silver ions) on cells’ gene expression pattern; and to investigate the molecular responses of fibroblast cells to different durations of exposure to electrical biostimulation and/or silver ions.

Another objective of this study was to determine which stimulus, the direct current or silver ion (or both), was responsible for the phenomenon. The great variety of applications of silver has been well documented since ancient times. Particularly, the antimicrobial action of silver compounds has been used in biomedical products since the 1700s [10, 11]. Currently, due to a consequence of increasing bacterial resistance to antibiotics, silver-containing antimicrobial agents have generated a renewed interest in the fields of skin infection management, burn healing, urinary catheters, and cardiovascular implants [12, 13]. The mechanisms of the antimicrobial action of silver ions have been summarized into three modes. Firstly, silver ions can bond with phospholipids and destabilize the cell membrane leading to loss of K^+^ ions and protons, and ultimately, cell death [14, 15]. Secondly, silver ions are known to interact with molecules inside the cell including amino acid residues, enzyme proteins, and nucleic acids, resulting in protein denaturation, inhibition of enzyme function, and DNA strand breakage and replication inhibition [16–18]. Lastly, the exposure to silver causes an increase of ROS production in the cell such as hydroxyl radical (•OH), hydrogen peroxide (H_2_O_2_), and superoxide radical (•O^−2^), which leads to oxidative stress and cell death [18–20]. Besides this literature focusing on the antimicrobial effects of silver ions, many questions regarding the biological effects of silver ions still remain. Moreover, the effects of silver ions on mammalian cells and the associated underlying mechanisms are still largely unknown, which generates concerns about its safety for applications in medical products and clinical settings [13, 15]. In order to explore the role of silver ions in the cellular events that occur upon exposure to Becker’s electrical biostimulation treatment, we conducted the present study that focused on relatively low levels of silver ion which is more in line with the situation in a clinical setting where a lower silver concentration is usually applied as an antibacterial agent [13, 21, 22]. Here, we detail our efforts to evaluate the relationship between cell viability and cellular morphological change in response to silver ion exposure, to analyze whether the time is a factor of inducing silver ion cytotoxicity in mammalian cells, to study the effects of silver ions on cellular metabolism by determination of levels of cellular metabolites such as ATP, NAD^+^/NADH, and ROS.

## Methods and materials

### Electrical biostimulation

**Fig 1:**
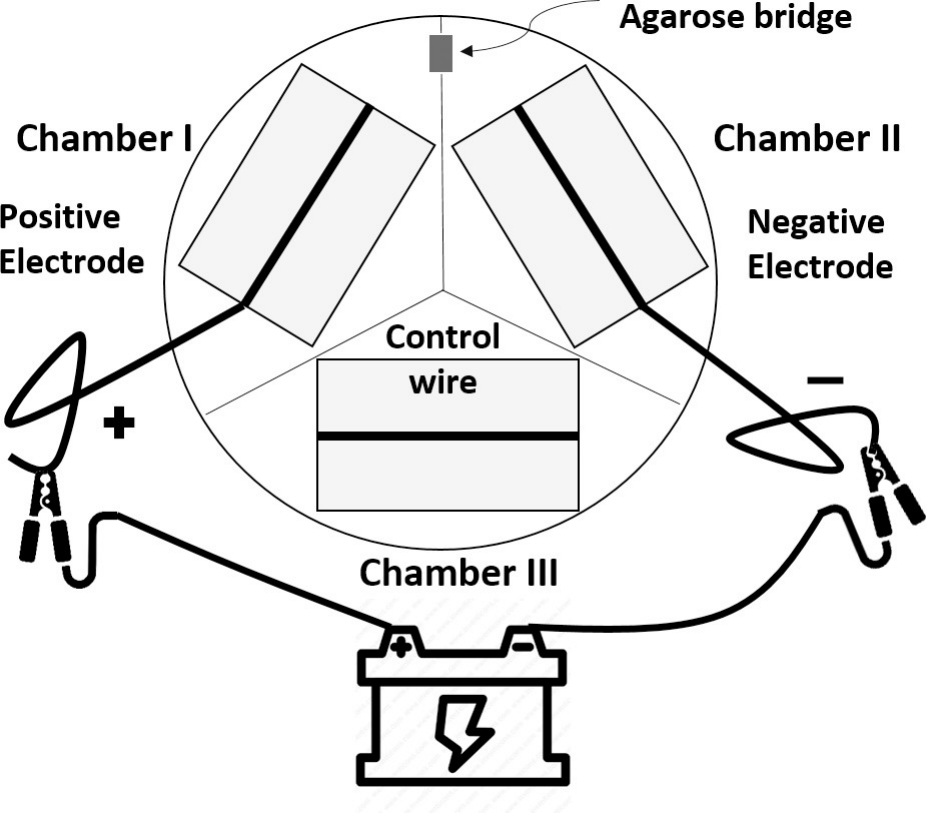
Diagram of the experimental apparatus of the electrical biostimulation treatment. Silver wires were affixed on microscope slides and placed into a three-chambered plastic petri dish. A power source was connected by the silver wires of Chamber I and Chamber II. Chamber I: the positive electrode (the anode); Chamber II: the negative electrode (the cathode); Chamber III: silver wire with no current passing (the vehicle control). Chamber I and Chamber II was bridged by an agarose gel to complete the circuit.

Porcine fetal-derived fibroblast cells were isolated, cultured in vitro, and passaged as described elsewhere [23]. The setup of the apparatus for electrical treatment is shown in Fig 1. Poly-L-Lysine coated microscope slides were cut (3.8 cm long and 2.5 cm wide) so as to fit flat in the bottom of a chamber of a three-chambered Y-segmented plastic 100 mm petri dish (FB087583; Fisher Scientific, Pittsburgh, PA). Twelve centimeters of 0.5 mm diameter 99.99% pure elemental silver wire (Cat #265586-10G, Sigma-Aldrich, St. Louis. MO) was affixed on each microscope slide with drops of RTV 118 High Performance Adhesive (Momentive Performance products, Waterford, NY). Approximately 0.7x10^6^ cells were plated into each chamber of the segmented plate and cultured with 6 mL of culture medium. The fibroblast culture medium contains Dulbecco’s Modified Eagle Medium (DMEM, 85% v:v), Fetal Bovine Serum (15% v:v), 2ng/mL basic Fibroblast Growth Factor (Sigma-Aldrich, MO, USA), and 20 μg/mL gentamicin (Corning, NY). Cells were incubated at 39°C, 100% humidity, and in a gas mixture of 6% CO_2_ in the air. The cells were prepared to be exposed to electrical current when reaching 80%-90% confluency. A B2902A source/measure unit (SMU; Agilent Technologies, Santa Clara, CA) produced and channeled the direct current electricity. The SMU lead “High”/ lead “Low” (positive/ negative side) was connected to the silver wire in Chamber I/ Chamber II of the plate respectively, while the wire in Chamber III was left unattached and served as a no-current control chamber in all experiments. An agar-salt bridge made of 3% (w:v) agarose in DMEM was positioned between Chambers I and II so as to complete the circuit. The SMU was programmed to provide constant current (i.e. 380 nA) through the system with approximately 0.35V of a total voltage, with electrons flowing in the direction of “high” to “low”. After 21 hours, cells were harvested from the chambers by scraping the cells off the glass slide with a pipette tip. The collected cells were centrifuged (450g for 5 minutes) to remove the supernatant medium and then snap-frozen at -80°C until use. These treatments were repeated three additional times on different days, for a total of four experimental replicates.

For the current intensity experiment, cells were exposed to direct current at intensities of 180 nA, 380 nA, and 580 nA for 21 hours. For the time of exposure experiment, cells were exposed to 380 nA direct current electricity for 5, 21, and 48 hours, and cells adjacent to the electrode were harvested. For the spatial location experiment, cells were treated with 380 nA direct current for 21 hours and then collected from different spatial locations within the treatment chambers: immediately adjacent to the silver electrode (< 5mm), 5–10 mm distal from the electrode, and 15–20 mm distal from the electrode.

Silver ion medium was prepared in the same manner as described above, except not seeding cells on the glass slide in the plastic petri dish. After 21 hours of exposure to the direct current, silver ion medium was collected from each chamber of the Y segmented plate and mixed well by pipetting up and down. Immediately, 500 μL of silver ion medium from each chamber was transferred to each well of a 4-well plate with cells at 80–90% confluency. After being treated with silver ion medium for 21 hours, cells were harvested and frozen as described above.

### RNA isolation and reverse transcription

Total RNA was extracted from the frozen cells using the Total RNA Kit I (OmegaBio-Tek Inc.; Norcross, GA, USA), according to the protocol recommended by the manufacturer. To confirm the quality and quantity of the extracted RNA, samples were analyzed by using the NanoDrop Spectrophotometer (Waltham, MA). For each cell sample collected, RNA was oligo-dT-primed to generate first-strand cDNA utilizing the GoScript Reverse Transcription (RT) System (Promega; Madison, WI). Then, cDNA quality was confirmed with a PCR reaction using Qiagen’s Hot Start PCR kit (Qiagen; Germantown, MD) and cDNA samples were stored at -20°C until further use.

### Fluidigm qPCR

Relative abundances of 48 transcripts were determined in each experimental sample using the 48.48 Dynamic Array Integrated Fluidic Circuit (IFC) from Fluidigm (South San Francisco, CA) for quantitative RT-PCR. Fluidigm’s recommended protocol (ADP14) was followed for gene expression analysis, which was described in detail previously [24]. Forty-eight genes were evaluated and grouped into one of eight functional categories: housekeeping (*EIF4A1*, *GAPDH*, *HSP90AA1*, *RPN1*, *TAF11*, *HPRT1*), apoptosis (*ATM*, *BCL2L1*, *CASP3*, *TP53*, *XIAP*), epigenetic modifiers (*ASH2L*, *DMAP1*, *DNMT1*, *DNMT3A*, *DNMT3B*, *EHMT2*, *EZH2*, *HDAC3*, *SIRT1*), potentially imprinted (*GNAS*, *GRB10*, *IGF2*, *IGF2R*, *NDN*, *NNAT*, *PEG10*, *UBE3A*), maternal effect (*BMP15*, *GDF9*, *MOS*, *NOBOX*, *ZAR1*, *ZP3*), pluripotency (*KLF4*, *LIN28*, *MYC*, *NANOG*, *POU5F1*, *SOX2*), sexing (*SRY*), and epithelial cell related (*ASCL2*, *CYP17A1*, *ELF5*, *HAND1*, *HSD17B1*, *KRT8*, *TEAD4*). Primer sets (see S1 Table for primer sequences) had been comprehensively validated to have similar amplification efficiencies previously [25].

### Live cell imaging and cell viability

Live/dead cell imaging was analyzed using the ReadyProbes Cell Viability Imaging Blue/Red kit (Life Technologies, Carlsbad, CA) according to the manufacturer’s instructions. After electrical biostimulation treatment, cells were stained with 2 drops (about 50 μl) per mL of the culture medium of each NucBlue Live reagent (Hoechst 33342) and propidium iodide and incubated for 15 minutes at room temperature. Images were obtained utilizing an inverted Zeiss microscope equipped for fluorescence detection.

Cell viability was assessed using the Countess automated cell counter for cell counting with the 0.4% trypan blue stain (Invitrogen, Carlsbad, CA). Briefly, after electrical biostimulation treatment, cells were collected and centrifuged. The culture medium was removed and cells were resuspended in 100 μl of fresh medium. Then 10 μl of the sample was mixed with 10 μl of trypan blue and pipetted into a Countess chamber slide for cell counting. The detailed procedure was followed according to the manufacturer’s instructions.

### Silver ion release measurements

Ion release from the silver wire was assessed using Thermo Scientific X-Series 2 inductively coupled plasma mass spectrometry (ICP-MS). The samples were digested in trace mineral grade nitric acid under heat at 90°C overnight. The digested solution was freshly diluted with ultra-pure water to a final nitric acid content of 5%, which provided a matrix match for the analytical standards. Before analysis, the ICP-MS was tuned using an aqueous multi-element standard solution (10 ng/mL each of Ba, Be, Bi, Ce, Co, In, Li, Ni, Pb, and U) for consistent sensitivity. The prepared samples were assessed against concentration curves of known silver standards. Standard curves and quality control samples were analyzed every 5 samples.

### RNA-sequencing

Total RNA from samples of silver ion medium treated cells were extracted as described above. cDNA synthesis and sequencing library preparation were performed by using KAPA stranded mRNA-Seq kit Illumina platform (Kapa Biosystems, Boston, MA). A detailed protocol was followed according to the manufacturer’s instruction (KR0960). Briefly summarizing, poly-A mRNA was first captured by using magnetic oligo-dT beads. The first strand cDNA was synthesized by random priming after poly-A mRNA was eluted from the beads and fragmented. The cDNA: RNA hybrids were converted to double-strand cDNA (dscDNA), which were marked by incorporating dUTP into, during the second strand synthesis. Then, by adding dAMP to the blunt ends (3’-ends) of the dscDNA, the fragments for adapter ligation were prepared. During adapter ligation, dsDNA adapters with 3’dTMP overhangs were ligated to the A-tailed DNA library insert fragments. Library fragments carrying adapter sequences at both ends were subjected to high-fidelity, low-bias PCR for enrichment. The strand marked with dUTP was not amplified which allowed strand-specific sequencing. The amplified library was purified by Agencourt AMPure XP beads (cat#A63881, Beckman Coulter Genomics, Chaska, MN) according to the manufacturer-recommended protocol. For quality control, libraries were quantified using the KAPA Library Quantification Kit for Illumina platforms (cat# KK4824). Assembled libraries and RNA samples were submitted to the University of Utah Huntsman Cancer Institute (salt lake city, UT) for RNA-sequencing (50 cycles single end read) using Illumina HiSeq2000 next-generation sequencer (San Diego, CA).

### Morphology analysis and cell viability

Cells were stained with 5 μg/mL Hoechst 33342 nucleic acid dye and imaged on an inverted Zeiss microscope after 21 hours of culture in silver ion medium. And immediately, the morphology and the total number of cells in the image were assessed by using the “Image Analysis” module for ZEN BLUE of Axiovision software (Carl Zeiss, AG, Germany). Hoechst stained fibroblast cells in the images were counted by the software representing the total cell number in the image. Fibroblast cells with rounded shape were considered to have morphological changes and were counted manually using bright field (transmitted light) images. Thus, the level of morphological change is presented in percentage where the number of cells with morphological change is over the total number of cells in the image (250 to 1000 total cells). Three images were analyzed for each sample and the average value was calculated for each sample. The experiment was repeated four times.

After silver ion medium treatment, cells were detached by trypsinization and then centrifuged. The culture medium was removed and resuspended in fresh medium. Immediately, cells were stained with 0.4% trypan blue solution and counted in a hemocytometer to assess the number of dead (blue) cells from the total number of cells counted.

### Quantification of cellular ATP

Silver ion treated-cells were transferred to an opaque white 96-well microplate and the total levels of cellular ATP (adenosine triphosphate) were determined using the Luminescent ATP Detection Assay Kit (Abcam, Cambridge, MA), according to the manufacturer’s instructions. The reaction of ATP with added firefly luciferase and luciferin generates light that is proportional to the ATP concentration inside the cell. Luminescence was measured by Synergy H1 Reader (BioTek, Winooski, VT) and the ATP concentration (pM/cell) was interpolated from an eight-point ATP standard curve. Experiments were repeated four times.

### NAD^+^ and NADH analysis

The NAD^+^ and NADH content of silver ion medium treated-cells was measured using a colorimetric NAD^+^/NADH ratio assay kit (AAT Bioquest, Sunnyvale, CA, USA) according to the manufacturer’s instruction. Briefly, cells were collected and resuspended with lysis buffer for 15 minutes. The lysate was centrifuged at 1500 rpm for 5 minutes and the supernatant was used for tests. The pool of NAD^+^ and NADH and the NAD^+^ extract were incubated with reaction mixture at room temperature, simultaneously. Three hours later, the absorbance at 460 nm was monitored by Synergy H1 Reader (BioTek, Winooski, VT). The amount of absorbance signal is directly proportional to the concentration of NAD^+^ and NADH, which is used as an indicator of the cellular NAD^+^ and NADH concentration. Experiments were repeated four times.

### Measurement of total ROS generation

To measure the level of intracellular reactive oxygen species (ROS), the ROS assay kit with 2’,7’-dichlorofluorescein diacetate (DCFDA) was utilized (ab113851, Abcam, Cambridge, MA). DCFDA is a cell-permeable fluorescent probe that can detect reactive oxygen species including hydroxyl, peroxyl radicals, and peroxynitrite. After silver ion medium treatment, cells were collected. After centrifugation, the pellet was resuspended in the diluted DCFDA solution provided by the kit. Cells were stained in the dark at 37°C for 45 minutes. DCFDA is deacetylated by cellular esterases to a non-fluorescent compound that cannot diffuse out of cells and can be oxidized by ROS into 2’,7’-dichlorofluorescein (DCF). DCF is a highly fluorescent compound that can be detected via excitation and emission spectroscopy at wavelengths of 485 and 535 nm respectively. The fluorescence intensity of intracellular DCF is a linear indicator of the amount of ROS activity in the cells. Experiments were repeated four times.

### Flow cytometry analysis

Cells were collected by trypsinization after treated with silver ion medium by 5hr, 21hr, and 48hr. Immediately, cells were stained with 7-aminoactinomycin D (7-AAD; Sigma Chemical Co., St. Louis, MO) and 20nM of SYTO 13 (Molecular Probes, Eugene, OR) in PBS for 30 minutes in the dark. 7-AAD is a cell impermeable DNA binding dye that excites at 488 nm and emits in the far red, with an emission peak at approx 670 nm. The cell-permeable SYTO 13 is a green fluorescent nucleic acid dye that excites at 488 nm and emits at 509 nm. Flow cytometry was performed with an Accuri C6 flow cytometer (BD Biosciences, San Jose, CA) that can detect SYTO 13 staining by FL1 (533/30 nM) and 7-AAD by FL3 (>670 nm), registered on a log scale. A threshold of channel 400,000 on FSC-H was set to gate out light scatter and fluorescence signals caused by debris in samples. The experiment was repeated four times with non-stained control. Samples were analyzed as: live normal cells–positive stain for SYTO 13; Necrotic cells–positive stain for SYTO 13 and 7-AAD.

### Data analysis

Raw qPCR data (C_T_ values) were converted to fold-change values (2^-ΔΔCT^) for comparison between sample types using the ΔΔC_T_ values method via the Fluidigm Real-Time PCR Analysis Software. The untreated control cells (Chamber III–no current) were selected as the “calibrator sample” and the composite average of all six housekeeping genes was used as the reference gene. A logarithmic transformation (Log_2_) of fold change values was performed before submitting the values for least squares analysis of variance using the General Linear Models procedure of SAS with Tukey HSD post hoc test. Significance was assigned at P-values < 0.05.

One-way analysis of variance (ANOVA) was performed in experiments of ATP, NAD^+^ and NADH, and ROS analysis. For cell viability data, two-way ANOVA was performed. Tukey’s test was performed for multiple comparisons. Statistical significance was defined as P <0.05. The analysis was carried out by using Graphpad Prism 8 software.

For RNA-sequencing data, read count normalization and alignment was performed at the Center for Integrated Biosystems (Utah State University), by aligned individual reads to the Ensembl transcript database (Sscrofa11.1) using STAR-Aligner. The aligned reads of expressed genes were then counted by using HTSeq-count. Identification of differentially expressed genes between silver ion medium treated cells and non-treated cells (control) was determined by the DESeq package that is based on a negative binomial distribution model [26]. P-values of significantly differentially expressed genes between treated and control were adjusted using the Benjamini and Hochberg’s approach to control the false discovery rate at FDR 0.05. [27]. For Gene Ontology analysis, the differentially expressed genes were classified into groups based on functional similarity using the DAVID Gene Functional Classification tool [28].

## Results

**Fig 2:**
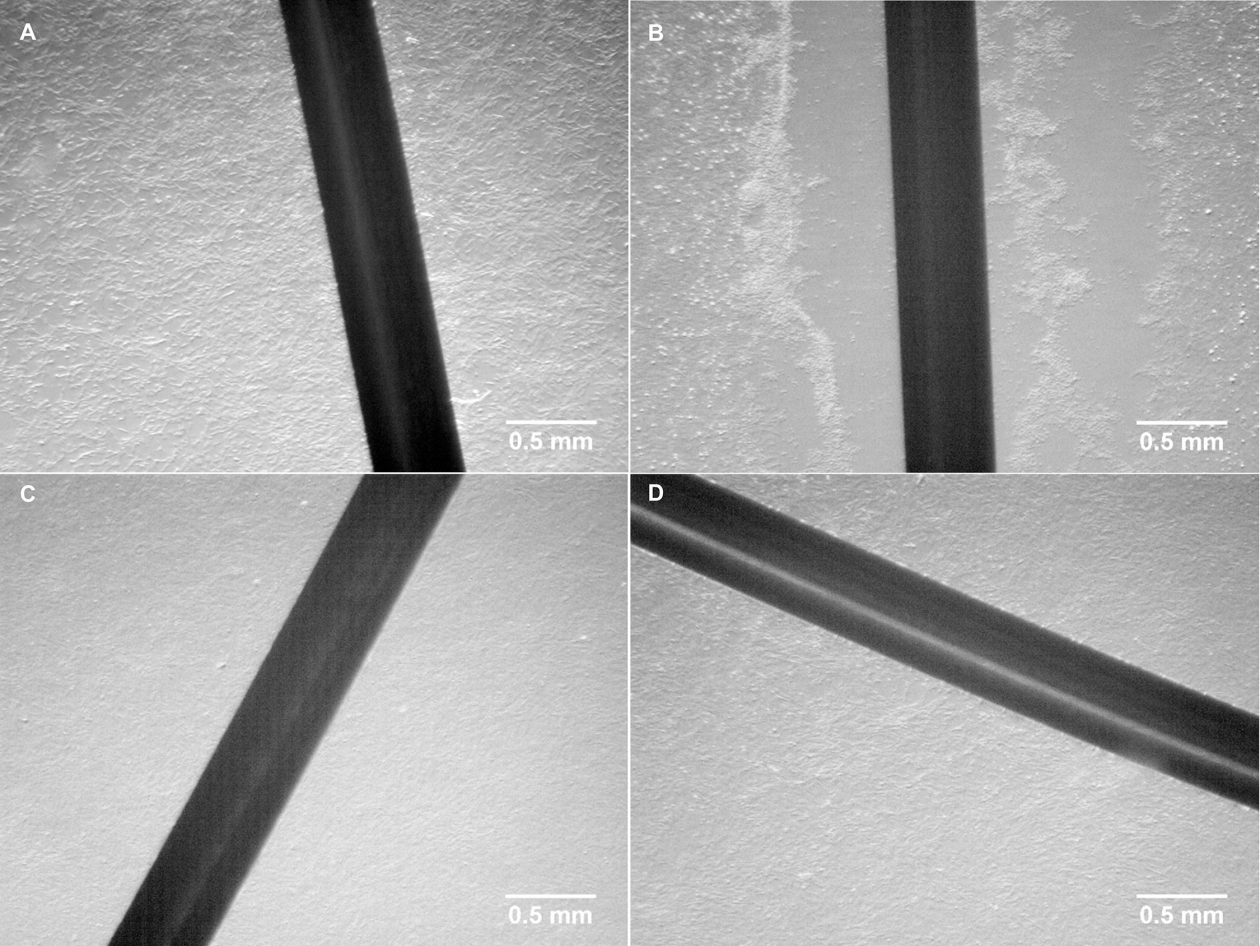
Visual appearance of cultured porcine fibroblast cells before and after treatment. **(A)** cells before electrical biostimulation; **(B)** cells in Chamber I (the anode) after exposed to electrical biostimulation (380 nA) for 21 hours; **(C)** cells in Chamber II (the cathode) after exposed to electrical biostimulation (380 nA) for 21 hours; **(D)** cells in Chamber III (no current as vehicle control) after exposed to electrical biostimulation (380 nA) for 21 hours.

In response to the 21 hours exposure to 380 nA electrical current and the presupposed concomitant increase in free silver cations in the Chamber I (the anode), cells in close proximity (i.e. within ~5 mm) to the silver electrode completely detached from the underlying substrate and could be easily picked up by gentle pipetting. From approximately 5 mm to 10 mm distal from the electrode, cells were mostly still attached, but somewhat loosely. The conglomeration of the cells was observed forming small clumps of estimated 3–100 cells, which were both attached and unattached and distributed in the space between 0–10 mm distal from the electrode. There was no morphological change observed in the cells beyond 10 mm distal from the electrode in Chamber I. Similarly, no phenotypic change was observed in the cells cultured in Chambers II or III. The results described above for the 21 hours exposure to 380 nA electrical current are depicted in Fig 2. All other exposure times or other intensities of current treatments had similar effects, except the morphology changed cells were observed at slightly different distances from the silver electrode.

To characterize in more depth the effects of electrical biostimulation on porcine fibroblast cells, gene expression analysis was performed using the microfluidics-based BioMark platform from Fluidigm (South San Francisco, CA) in three experiments: 1) current intensity; 2) time of exposure; 3) spatial locations. Note that, the gene products of *BMP15*, *CYP17A1*, *IGF2*, *SRY*, *DNMT3B*, *ELF5*, and *ZAR1* were undetectable and not included further in the data analyses.

**Fig 3:**
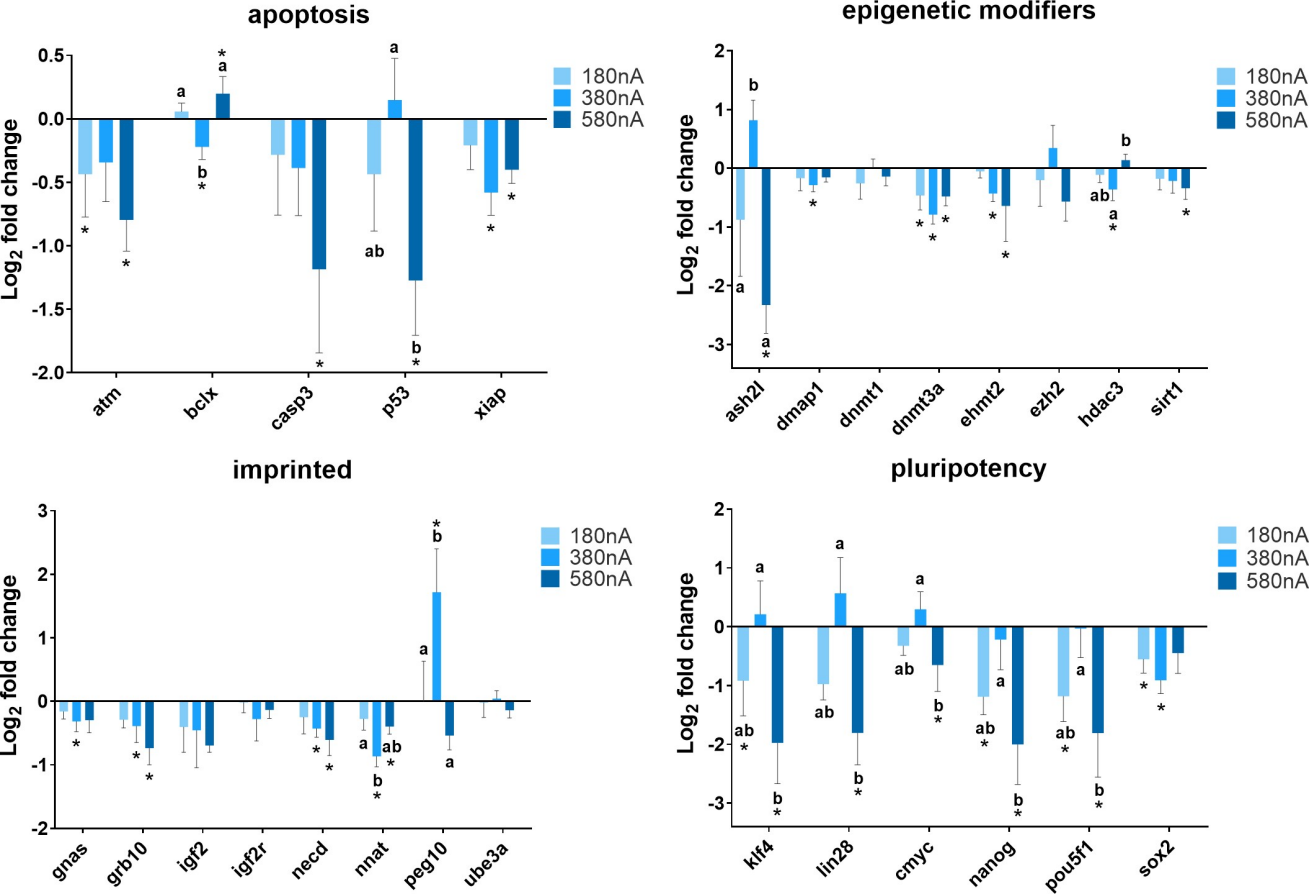
Summary of relative gene expression levels in fibroblast cells of Chamber I exposed to 180 nA, 380 nA, and 580 nA of electrical biostimulation for 21 hours. Graphs depicting the average log_2_ fold change values of every gene for each of the functional gene categories tested by qPCR using the BioMark system from Fluidigm. In each of these graphs, error bars reflect the standard error of the mean log_2_ fold change values calculated for four replicate reactions. * = significantly different from the control (the cells in Chamber III of the 180 nA, 380 nA, and 580 nA experiment, respectively). a,b,c = within a panel (gene category), different superscripts denote statistically significant differences in log_2_ fold change values between samples (P < 0.05).

In order to assess how the intensity of electrical biostimulation might influence patterns of gene expression, cells were exposed to direct electrical current channeled through the silver wire at intensities of 180 nA, 380 nA, and 580 nA for 21 hours. As shown in Fig 3, the expression of *ATM* decreased when cells were treated with 180 nA (P = 0.045) and 580 nA (P = 0.004) direct current compared to the control (Chamber III). *BCLX* gene expressed at a lower level when cells exposed to 380 nA of the direct current (P = 0.017), and 580 nA was significantly higher than the control (P = 0.029). When treated with 580 nA of the direct current, the expression of *CASP3* (P = 0.005) and *SIRT1* (P = 0.028) decreased compared to the control. The expression of *DMAP1* (P = 0.016) and *GNAS* (P = 0.032) was lower than the control when treated with 380 nA but not 180 nA and 580 nA of the direct current. Compared to the control, *XIAP* expression dropped markedly in cells exposed to 380 nA (P < 0.001) and 580 nA (P = 0.007), and there was no change in cells treated with 180 nA of the direct current (P = 0.079), as well as *EHMT2*, *GRB10*, and *NECD*. *ASH2L* was more highly expressed in cells shocked by 380 nA electrical current compared to 180 nA and 580 nA. For the *DNMT3A* gene, electrical biostimulation induced a significant decrease in its expression in cells at all three intensities (180 nA, P = 0.004, 380 nA, P < 0.001, 580 nA, P = 0.008). A relatively lower expression of *HDAC3* was observed in cells exposed to 380 nA current compared to the control (P = 0.005) and 580 nA (P = 0.025). Similarly, the expression of *NNAT* was much lower comparing cells exposed to 380 nA with 180 nA (P = 0.023) and the control (P < 0.001). *PEG10* gene showed significant upregulation in response to 380 nA electrical current. For the pluripotency gene category, a decrease of expression was observed in most of the cells after exposure to electrical biostimulation; and only when cells treated with 380 nA electrical current, *KLF4*, *LIN28*, *NANOG*, *POU5F1*, and *CMYC* tended to expressed somewhat more, but still at a similar level to the control. Additionally, this similar pattern of gene expression change was observed in *P53* and *ASH2L* as well.

**Fig 4:**
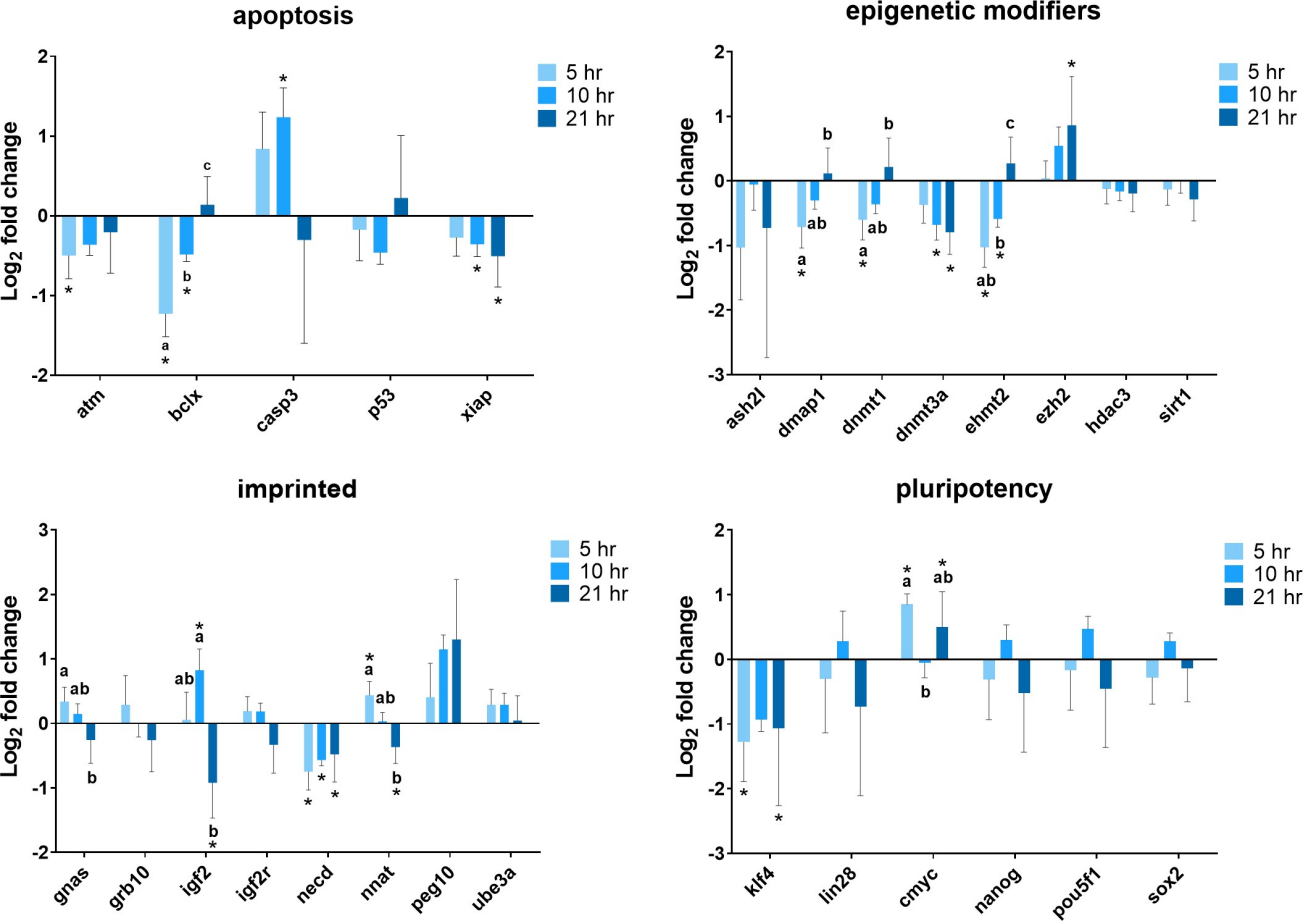
Summary of relative gene expression levels in fibroblast cells of Chamber I exposed 380 nA of electrical biostimulation for 5, 10, and 21 hours. Graphs depicting the average log_2_ fold change values of every gene for each of the functional gene categories tested by qPCR using the BioMark system from Fluidigm. In each of these graphs, error bars reflect the standard error of the mean log_2_ fold change values calculated for four replicate reactions. * = significantly different from the control (the cells in Chamber III of the 5, 10, and 21 hours experiment, respectively). a,b,c = within a panel (gene category), different superscripts denote statistically significant differences in log_2_ fold change values between samples (P < 0.05).

Gene expression analysis of cells exposed to 380 nA direct current electricity for 5, 10, and 21 hours was conducted to establish whether or not there is a time-dependent effect (Fig 4). The expression of *BCLX* (P < 0.001), *DMAP1* (P = 0.001), *DNMT1* (P = 0.006), and *EHMT2* (P < 0.001) genes dropped markedly after exposed to the electrical biostimulation for 5 hours, and recovered over time to a similar level as the control at 21 hours. *XIAP* gene expression decreased in the course of time with a significantly lower level to the control after cells exposed to electrical current for 10 hours (P = 0.043) and 21 hours (P = 0.006). The expression of *DNMT3A* tended to decrease, whereas *EZH2* tended to increase as time went by. *NNAT* expression was higher after 5 hours of exposure (P = 0.004) and reduced to a level similar to the control at 10 hours (P = 0.814), eventually decreased to a significantly lower level after 21 hours of exposure (P = 0.013). The expression of *GNAS* gene showed similar change patterns, except cells exposed for 5 hours (P = 0.054) and 21 hours (P = 0.134) were not different than the control as what *NNAT* gene was. The expression level of *IGF2* was much higher in cells at 10 hours (P = 0.013) but dropped notably at 21 hours (P = 0.006). A relatively low expression level of *NECD* was observed in cells at all three treatment time points (5 hours, P < 0.001, 10 hours, P = 0.005, 21 hours, P = 0.015). There were not a lot of changes with respect to pluripotency genes. After cells exposed to electrical current for 5 hours and 21 hours, *KLF4* expression decreased compared to the control (P = 0.015 and P = 0.037, respectively), and oppositely, the expression of *CMYC* was higher (P < 0.001 and P = 0.031, respectively).

**Fig 5:**
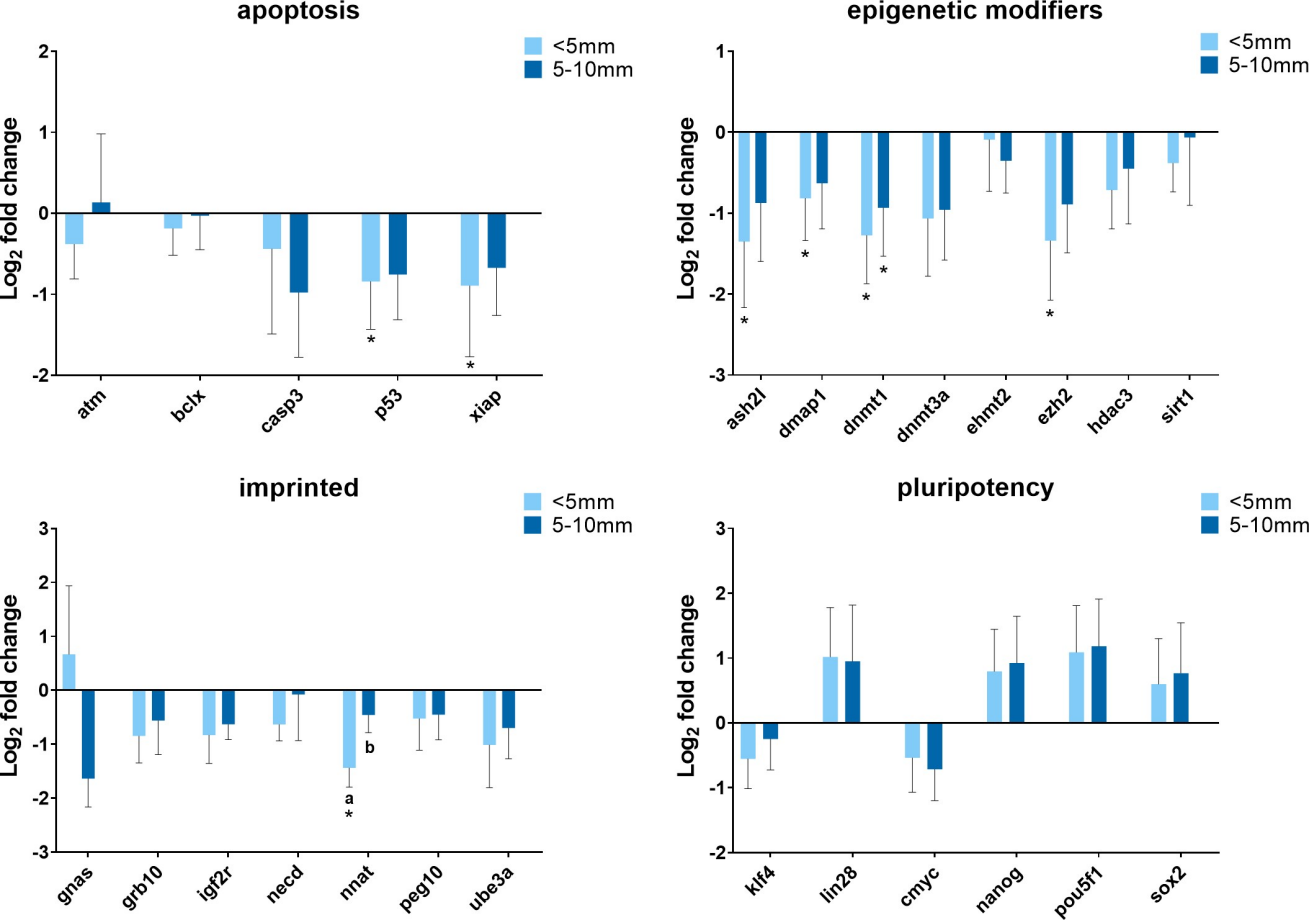
Summary of relative gene expression levels in fibroblast cells of Chamber I from different distal locations. Cells were exposed to electrical biostimulation (380 nA) for 21 hours, and collected from different locations: immediately adjacent (within 5 mm) to the silver electrode; 5–10 mm distal from the electrode; and 15–20 mm distal from the electrode (the control). Graphs depicting the average log_2_ fold change values of every gene for each of the functional gene categories tested by qPCR using the BioMark system from Fluidigm. In each of these graphs, error bars reflect the standard error of the mean log_2_ fold change values calculated for four replicate reactions. * = significantly different from the control (the cells in 15–20 mm distal from the electrode). a,b,c = within a panel (gene category), different superscripts denote statistically significant differences in log_2_ fold change values between samples (P < 0.05).

Since cells assumed very different morphological characteristics after treatment, it is necessary to determine whether the morphological and physiological changes (i.e. gene expression) of cells are coupled or independent of each other. Cells were exposed to 380 nA of direct electrical current for 21 hours and immediately collected from different spatial locations within the treatment chambers for gene expression analysis. The allocation of different spatial locations is described as below–close range: immediately adjacent (within 5 mm) to the silver electrode (obvious morphological changes including cell clumps); middle range: 5–10 mm distal from the electrode (less conspicuous morphological changes); and far range: 15-20mm distal from the electrode (no observable morphological changes). The qPCR data are presented in Fig 5 and cells from the far range served as the control. With respect to cells from different locations, most of the genes were expressed at similar levels with several exceptions. For example, *NNAT* gene was much less expressed in cells at the location adjacent to the silver electrode compared with cells from the middle range (P = 0.041) and the far range (P = 0.003). The expression of *P53*, *XIAP*, *ASH2L*, *DMAP1*, and *EZH2* were decreased in cells close to the electrode when comparing to cells that were far from the electrode, while the expression of these genes was also decreased in cells from the middle range but was not significantly different from the control. As cells were closer to the silver electrode, *DNMT1* gene expression was reduced in a distance-dependent manner. There was a trend that some pluripotency genes were up-regulated when cells were closer to the electrode (close and middle range) but not statistically different from the control. Generally, cells located closer to the electrode tended to have more changes in gene expression.

**Fig 6:**
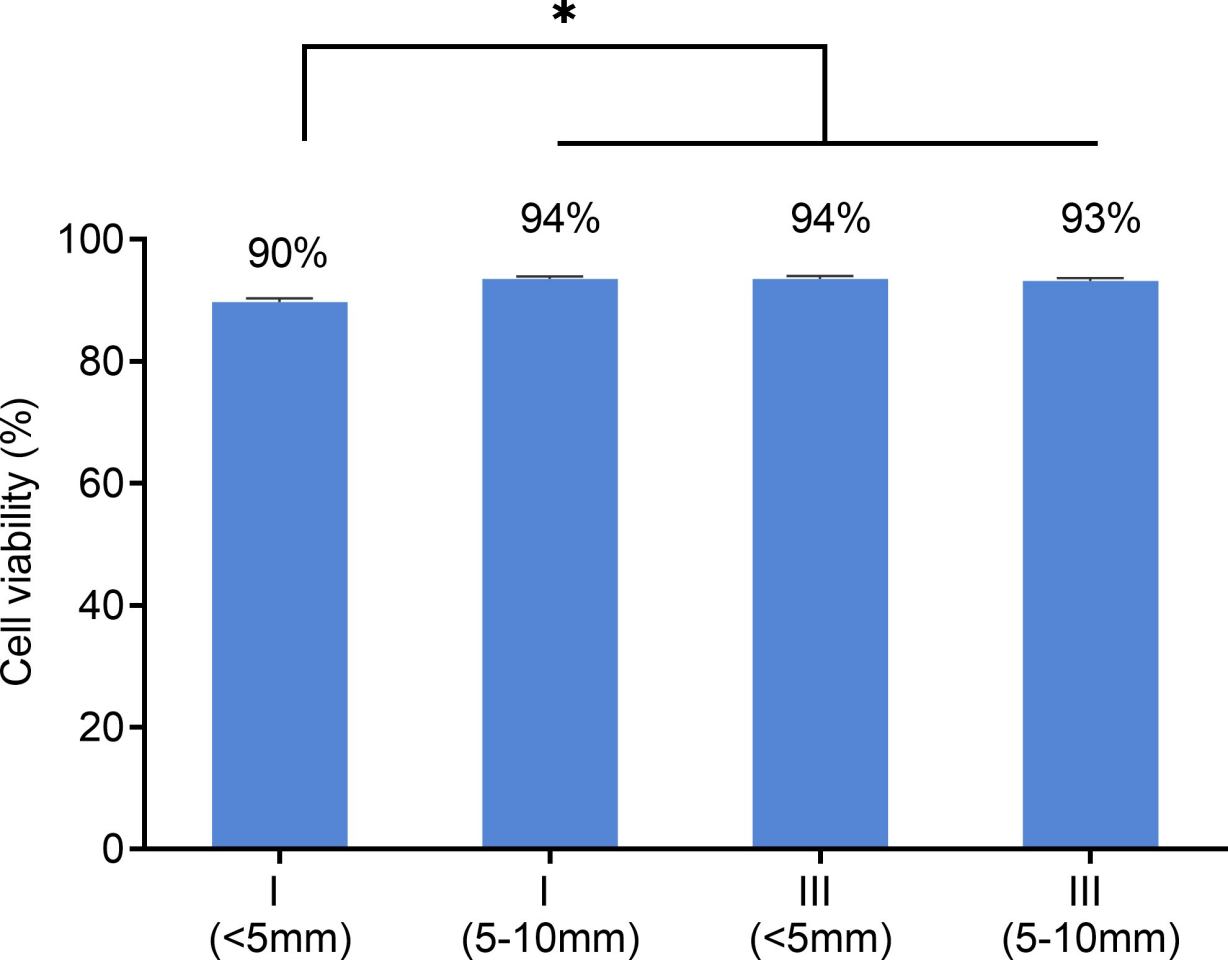
Effects of electrical biostimulation on fibroblast cells viability. After 21 hours of exposure to 380 nA of direct current and concomitant silver ions, the viability of cells from the locations immediately adjacent to the silver electrode (< 5mm) and 5–10 mm distal from the electrode of Chamber I and III respectively was assessed using countess automated cell counter. Data are presented as mean ± error of 4 independent replicates. * = significantly different between samples.

**Fig 7:**
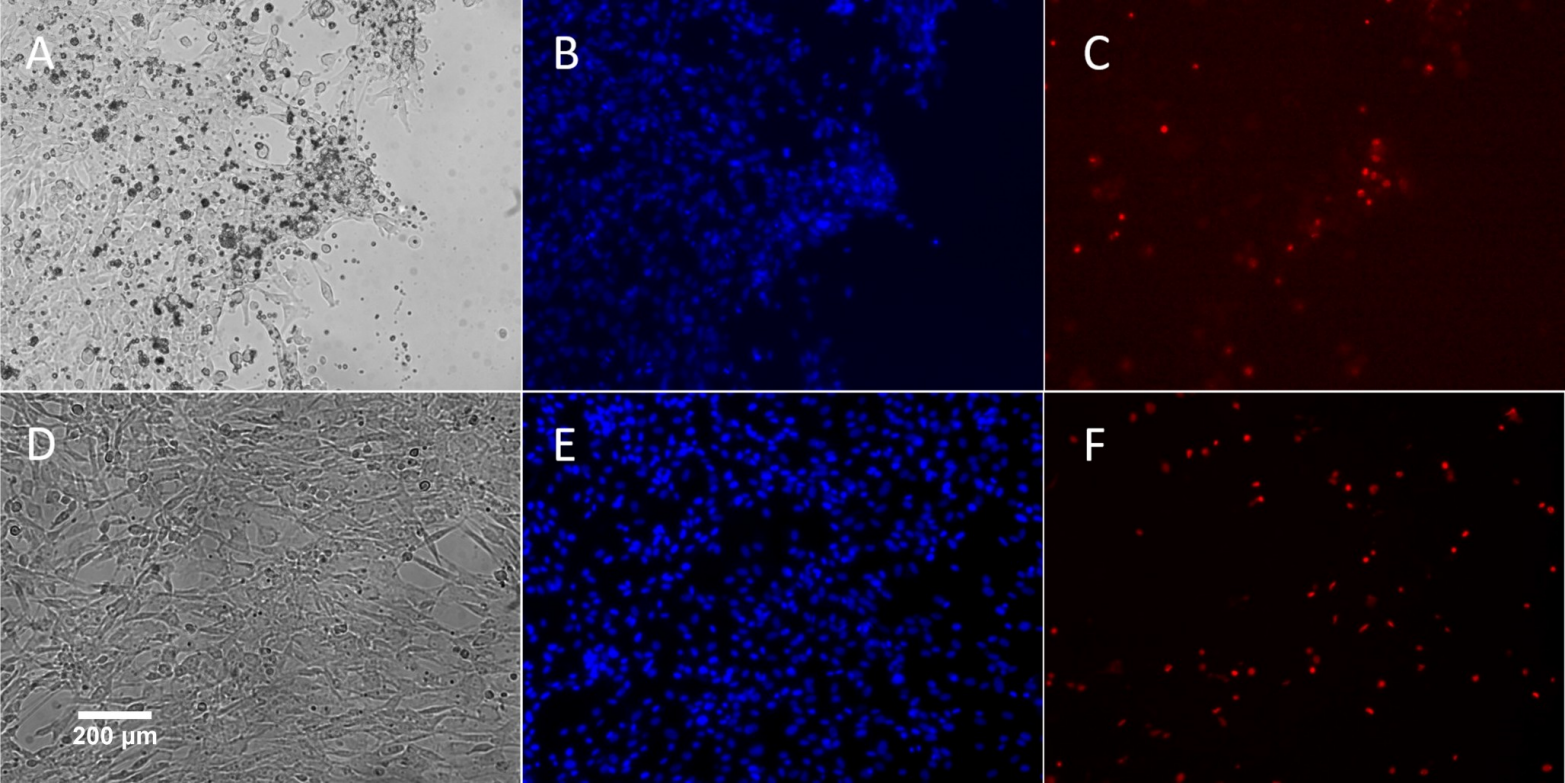
A representative live cell image of porcine fibroblast cells after exposure to 380 nA of electrical biostimulation. **(A-C)**: Same cells in Chamber I; **(D-E)**: Same cells in Chamber III. **(A)** and **(D)**: Cells visualized with transmitted light. **(B)** and **(E)**: Blue fluorescence represents Hoechst 33342 nuclear staining. **(C)** and **(F)**: Red fluorescence represents staining with propidium iodide.

The effect of the electrical biostimulation on the viability of the cells was determined using the Countess automated cell counter (Invitrogen, Carlsbad, CA). Cells were collected from close and middle range (as described above) within Chamber I and III after exposed to 380 nA of direct electrical current for 21 hr. As shown in Fig 6, though there was a slight decrease in viability of cells adjacent to the electrode in Chamber I. The majority of cells from Chamber I, despite the locations, were alive after the treatment (90% viability for location A, 94% for location B). In comparison, cells from the control Chamber III have approximately 93% to 94% of viability rates. To further verify the results, treated cells were stained with Hoechst and propidium iodide for live/dead cell imaging analysis using the ReadyProbes Cell Viability Imaging Blue/Red kit. In agreement with the result of the Countess cell counter, the fluorescent staining (Fig 7) also shows the majority of treated cells from Chamber I was alive.

**Fig 8:**
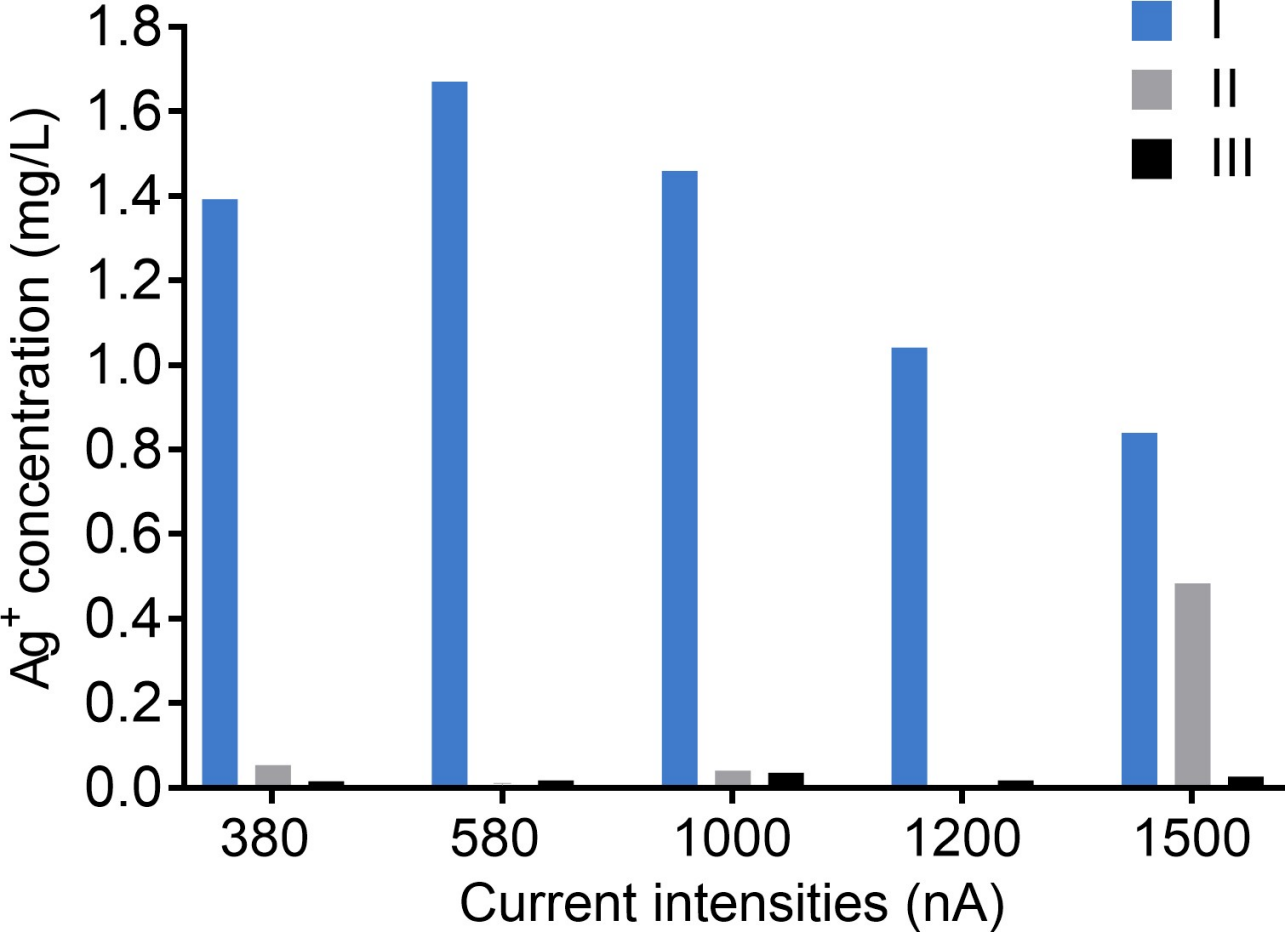
Silver ion concentrations in the cell culture medium generated by different intensities of electrical current for 21 hours. Silver ions in medium from Chamber I (blue bar), Chamber II (grey bar), and Chamber III (black bar) were measured by using inductively coupled plasma mass spectrometry (ICP-MS). The experiment was performed once.

In the theory of electrochemistry, when oxidation occurs at the anode, the electrode losses electrons, and the cation is released [22]. Therefore, the release of silver ions from Chamber I (the anode) was assessed by measuring the concentration of silver ions in the cell culture medium after direct current was applied for 21 hours by using ICP-MS (Fig 8). As expected, a significantly higher amount of silver ions was produced in Chamber I compared to the negligible concentration of silver ions in Chamber II and III. However, the increase of current intensity did not release more silver ions into the cell culture medium. This was probably due to a lack of replicates and some issues with the measurement process such as sample degradation or sample collection errors. The highest concentration of silver ions in the medium was detected when 580 nA of direct current was applied. Overall, as these data proved, it is important to note that the term “electrical biostimulation” we described in this study should include at least two factors without repeated explanation, which are the electromagnetic field and the electrically generated silver ions.

In order to conduct a more consistent experiment and limit the variance of results, we decided to change the treatment method from directly exposing cells to the electrical current and the silver ions concurrently to culturing cells with silver ion conditioned medium (see Methods for details). Moreover, based on the results we found, it is clear that the silver ion was mainly responsible for the observed changes in the cells but not the electrical current. The reason is that only cells in Chamber I assumed obvious morphological changes but not in Chamber II, in which the same intensity of electrical current also flowed through.

**Fig 9:**
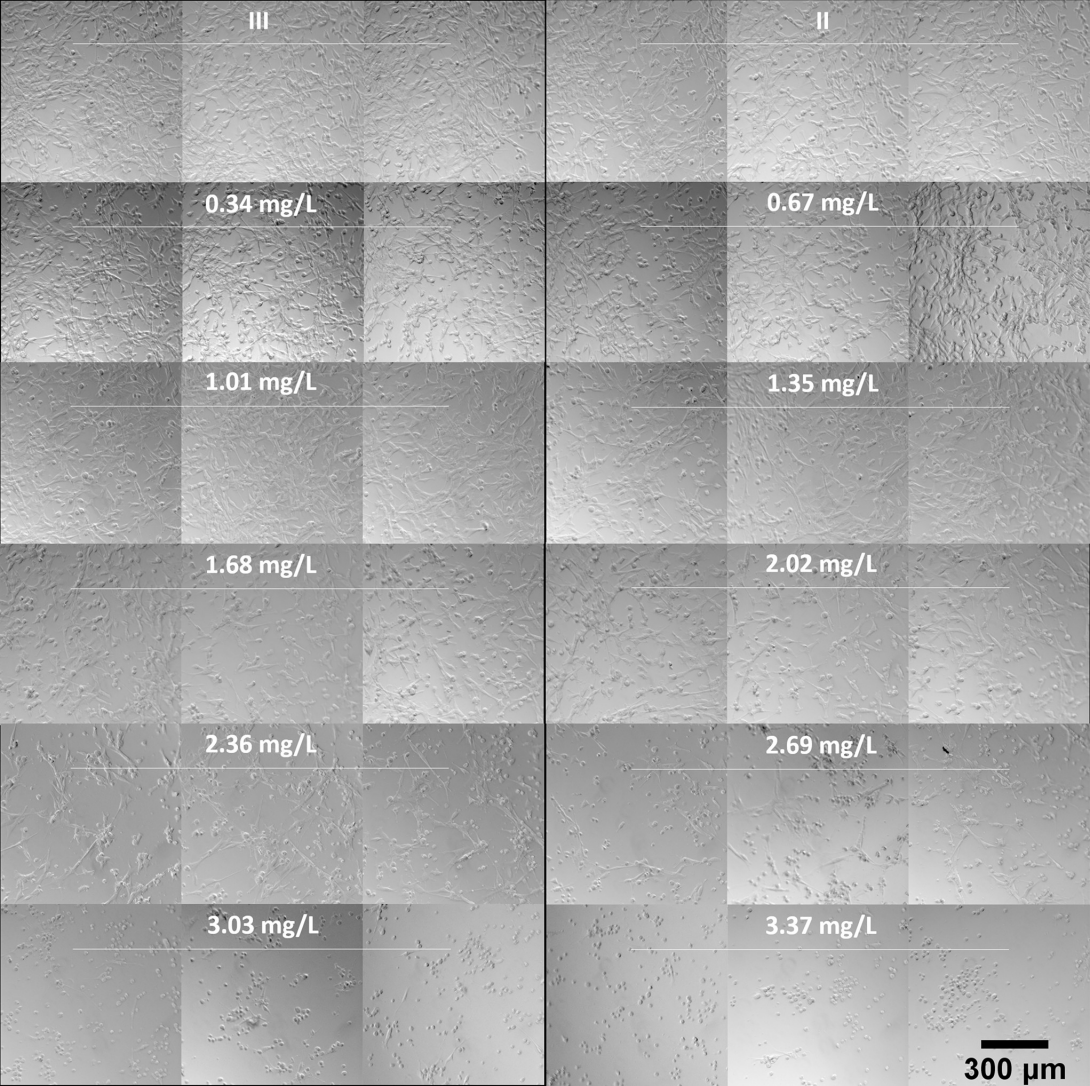
Representative images of the visual appearance of cultured porcine fibroblast cells exposed to electrically generated silver ions. 3.37 mg/L of silver ions were generated by 380 nA of direct current from pure silver electrodes. A series of 10% dilution of silver ion medium was prepared and immediately transfer to fibroblast cells for 21 hours of incubation. Chamber II (the cathode) and III (no current) are controls (no silver ions).

Normal fibroblast cells on the usual flat tissue culture substrate have the well-known elongated and flattened oblong or triangular shape with extended borders making contact with their neighbors [29]. To study the effects of electrically generated silver ions on porcine fibroblast cell morphology, a series of 10% medium dilutions was made from a working stock of silver ion-conditioned medium (3.37 mg/L of silver ion) to treat cells for 21 hours. Cells that were cultured with medium containing no silver ion (a negligible level) from Chamber II and Chamber III served as controls with normal fibroblast morphology. In response to silver ions, obvious morphological changes were found in cells that lost their original extended shape and exhibited a rounded form. As shown in Fig 9, when treated with relatively higher levels of silver ion, 3.37 and 3.03 mg/L, cells found all over the well were rounded and tended to have less adhesion with their neighbors and detached from the substrate. Some cells treated with 2.69 and 2.36 mg/L of silver ions retained their normal fibroblast shape, and the rounded cells remained attached to the substrate and had more adhesion with nearby cells, forming clumps. For cells treated with 2.02 and 1.68 mg/L, they did not elongate as long as normal fibroblast cells do. Lower concentrations of silver ions including 1.35, 1.01, 0.67, and 0.34 mg/L had no effects on the morphology of fibroblast cells. We also observed that there were much fewer cells in medium with relatively moderate and higher levels of silver ions (more than 1.68 mg/L). These results indicated that silver ions can affect cell morphology as well as cell proliferation.

**Fig 10:**
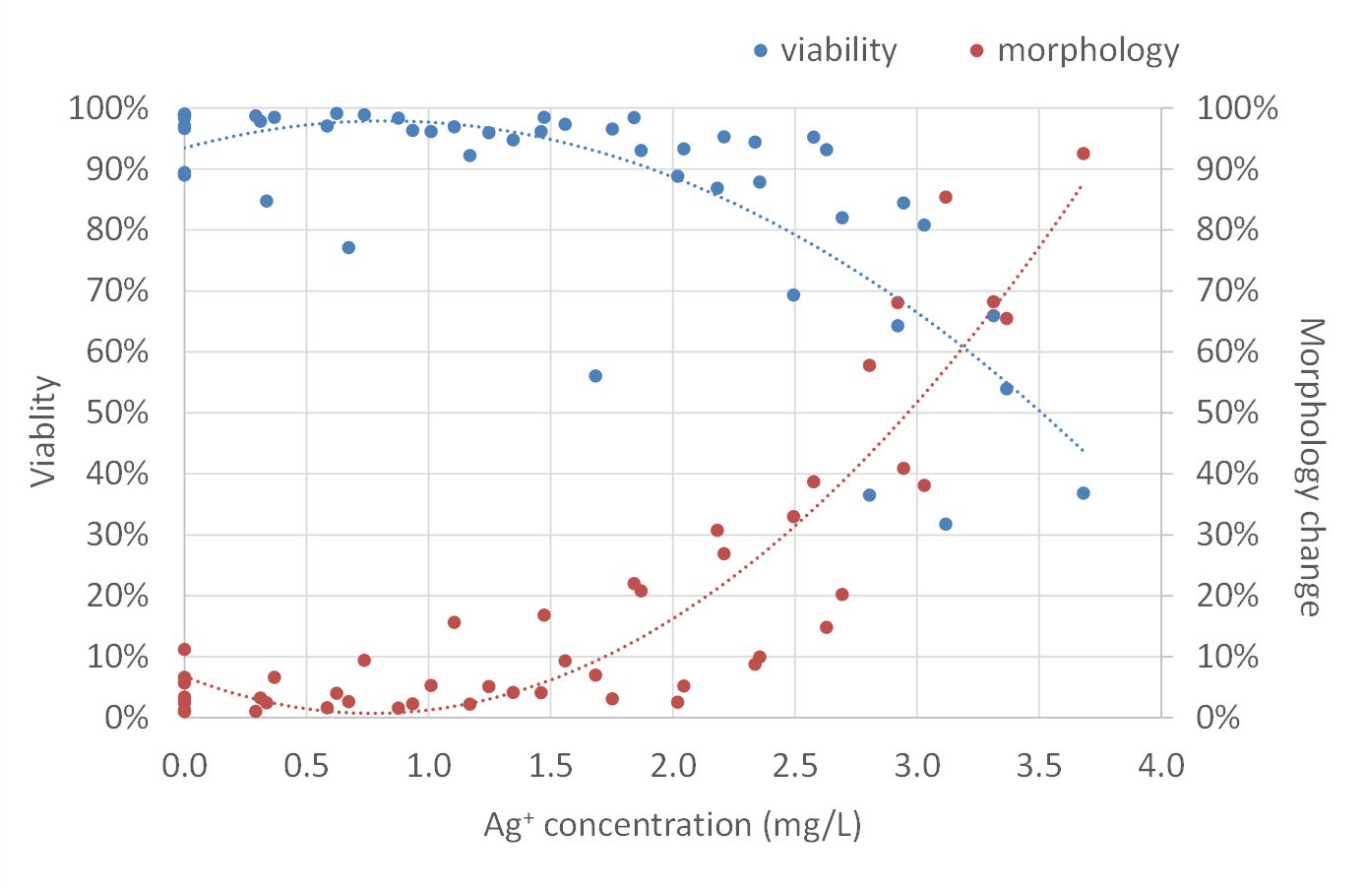
Correlation of fibroblast cell viability and morphology change in response to different concentrations of electrically generated silver ions. Cell viability (blue dots) was analyzed by trypan blue staining. Morphology change (red dots) was assessed by the “Image Analysis” module of Axiovision software. Polynomial trendlines of cell viability and morphology change are shown in blue dot line and red dot line, respectively. Silver ion concentration (mg/L) was determined by ICP-MS. Experiments were repeated four times.

To further study cell morphological change in response to electrically generated silver ions, as well as the relationship between cell morphology and cell viability, we assessed cell morphology change by Axiovision software in cooperation with Hoechst 33342 nucleic acid staining and determined cell viability using Trypan blue cell counting. As shown in Fig 10, silver ions did not affect cell viability and cell morphology below the concentration of 1.50 mg/L. Beyond that point, as silver ion concentration increased, cell viability went down and more cells showed morphological changes. With 2.00 mg/L of silver ions, approximately 90% of cells were alive and 15% of cells changed morphology. Cells treated with 2.50 mg/L of silver ions had decreased cell viability (80%), with approximately 30% of cells showing altered morphology. Cell viability dropped rapidly when treating cells with silver ions more than 2.50 mg/L indicating a toxic effect of electrically generated silver ions on mammalian cells at this level. These results suggested that not all of the cells with morphological changes were dead and some live cells also exhibited a change in morphology. These findings were further supported by recovering cells with morphological changes: after treatment, the rounded-up cells were immediately picked up by gentle pipetting and transferred into the normal fibroblast culture medium for a period of time (3–5 days), the cells then returned to the elongated shape, grew, and proliferated as normal fibroblasts (not shown).

To unravel the cellular functions affected by exposure to electrically generated silver ions, we performed gene expression analysis using RNA-sequencing for cells cultured with the silver ion conditioned medium for 21 hours. The medium was made by exposure with 580 nA of direct current for 21 hours (see Methods for details). Between Chamber I and Chamber III, 67 transcripts were found to be differentially expressed at FDR 0.05 (S2 Table). Of these transcripts, 63 were more abundant and 4 were down-regulated in Chamber I. Gene ontology analysis was performed by Gene Functional Classification Tool, which classified the 67 transcripts list into functionally related gene groups. Table 1 shows the list of the significantly enriched biological processes, in which we found the majority of them are associated with cellular metabolism. More specifically, respiratory chain, electron transport, oxidative phosphorylation, and ATP synthesis are all fundamental components of cellular metabolism and mitochondria function. No genes related to pluripotency or cellular reprogramming were found expressed differently between Chamber I and Chamber III.

**Table 1:** Gene set enrichment analysis using DAVID/EASE functional annotation tool.

Database	Term	Adjusted P-value	Genes
UP_KEYWORDS	Respiratory chain	0.0000	ND1, ND2, ND3, ND4, ND4L, ND5, ND6, CYTB, COX1, COX2
UP_KEYWORDS	Ubiquinone	0.0000	ND1, ND2, ND3, ND4, ND4L, ND5, ND6, CYTB
UP_KEYWORDS	Electron transport	0.0000	ND1, ND2, ND3, ND4, ND4L, ND5, ND6, CYTB, COX1, COX2
KEGG_PATHWAY	Oxidative phosphorylation	0.0000	ATP6, ATP8, ND1, ND2, ND3, ND4, ND4L, ND5, ND6, CYTB, COX1, COX2, COX3
KEGG_PATHWAY	Parkinson’s disease	0.0000	ATP6, ATP8, ND1, ND2, ND3, ND4, ND4L, ND5, ND6, CYTB, COX1, COX2, COX3
UP_KEYWORDS	Mitochondrion	0.0000	TMEM173, ATP6, ATP8, ND1, ND2, ND3, ND4, ND4L, ND5, ND6, CYTB, COX1, COX2, COX3
GOTERM_CC_DIRECT	Respiratory chain	0.0000	ND2, ND3, ND5, ND6, COX1, COX2
GOTERM_MF_DIRECT	NADH dehydrogenase (ubiquinone) activity	0.0000	ND1, ND2, ND3, ND4, ND4L, ND5, ND6
UP_KEYWORDS	Mitochondrion inner membrane	0.0000	ATP6, ND1, ND2, ND5, CYTB, COX1, COX2, COX3
UP_KEYWORDS	NAD	0.0000	ND1, ND2, ND3, ND4, ND4L, ND5, ND6
GOTERM_CC_DIRECT	Mitochondrial respiratory chain complex I	0.0000	ND1, ND2, ND3, ND4, ND4L, ND5
UP_KEYWORDS	Transport	0.0000	ATP6, ATP8, ND1, ND2, ND3, ND4, ND4L, ND5, ND6, CYTB, COX1, COX2,
GOTERM_CC_DIRECT	Mitochondrial inner membrane	0.0000	ATP6, ND1, ND2, ND5, CYTB, COX1, COX2, COX3
UP_KEYWORDS	Oxidoreductase	0.0000	ND1, ND2, ND3, ND4, ND4L, ND5, ND6, COX1
GOTERM_BP_DIRECT	ATP synthesis coupled electron transport	0.0001	ND4, ND4L, ND5, COX2
GOTERM_CC_DIRECT	Respiratory chain complex IV	0.0012	COX1, COX2, COX3
INTERPRO	NADH	0.0039	ND2, ND4, ND5
UP_SEQ_FEATURE	Transmembrane region	0.0056	ATP6, ATP8, ND1, ND2, ND3, ND4, ND4L, ND5, ND6, CYTB, COX1, COX2, COX3
KEGG_PATHWAY	Metabolic pathways	0.0071	GOT1, RDH12, ATP6, ATP8, ND1, ND2, ND3, ND4, ND4L, ND5, ND6, CYTB, COX1, COX2, COX3
KEGG_PATHWAY	Alzheimer’s disease	0.0170	ATP6, ATP8, CYTB, COX1, COX2, COX3
KEGG_PATHWAY	Huntington’s disease	0.0230	ATP6, ATP8, CYTB, COX1, COX2, COX3
UP_KEYWORDS	Acetylation	0.0400	ATP8, GPN2, CRYAB, HSP70.2, MT2B, NEFM
KEGG_PATHWAY	Cardiac muscle contraction	0.0420	CYTB, COX1, COX2, COX3

**Fig 11:**
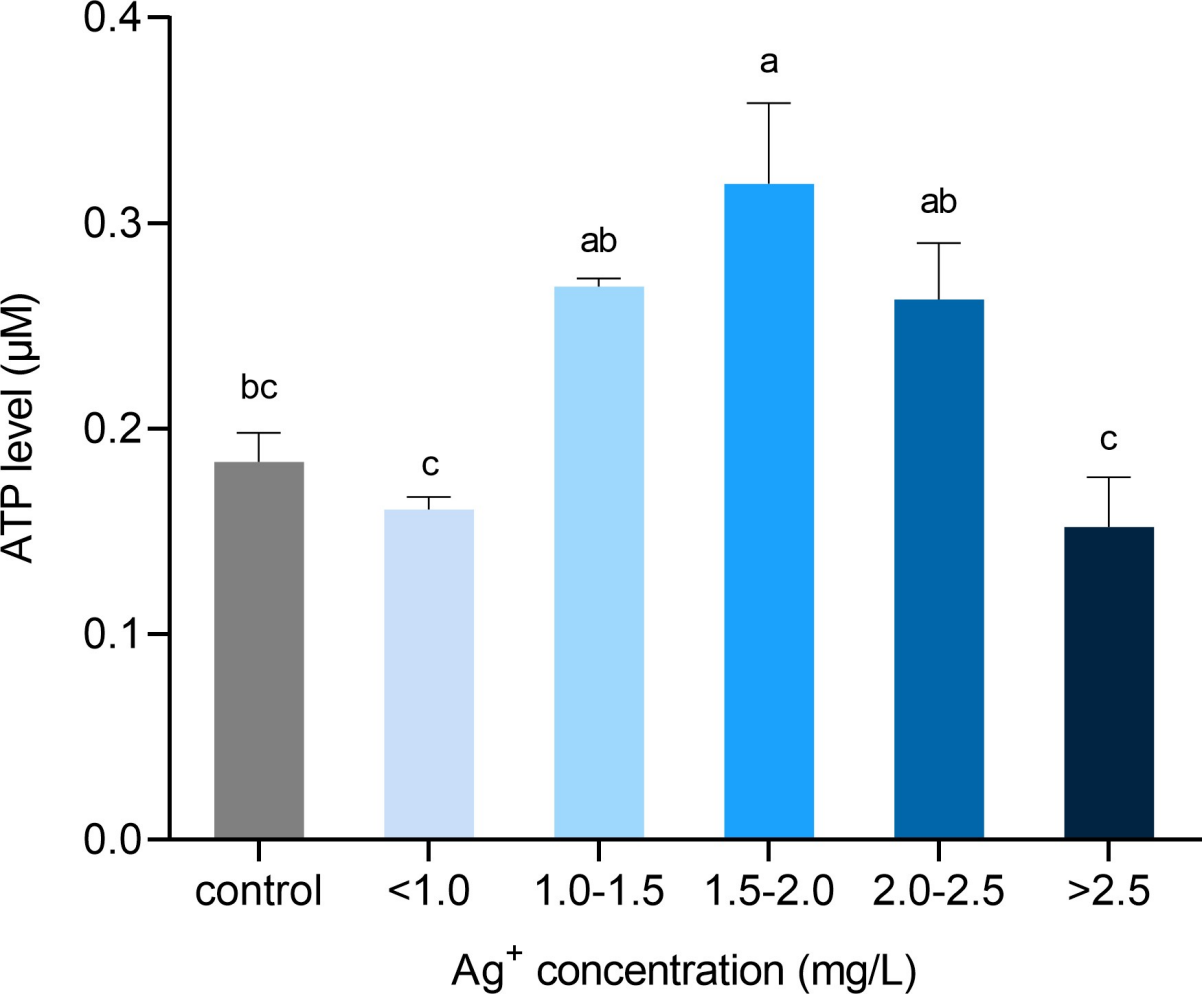
Quantification of intracellular ATP content from cells exposed to different concentrations of silver ions for 21 hours. Calculation of cellular ATP by interpolating standard curve values in a luminescent microplate assay. Values were normalized to living cell number. Means ± SEM. a, b, c, d = different superscripts denote statistically significant differences in ATP values between samples (P < 0.05). Experiments were repeated four times.

To study the effects of electrically generated silver ions on cell metabolism, we measured intracellular ATP level in porcine fibroblasts by a luminescent assay. ATP production was significantly increased in cells treated with 1.50 to 2.00 mg/L of silver ions (Fig 11). Beyond this point, as silver ion concentration increased, cells generated less ATP. When cells were cultured with more than 2.50 mg/L of silver ions, their ATP production dropped to a level that was similar to the control.

**Fig 12:**
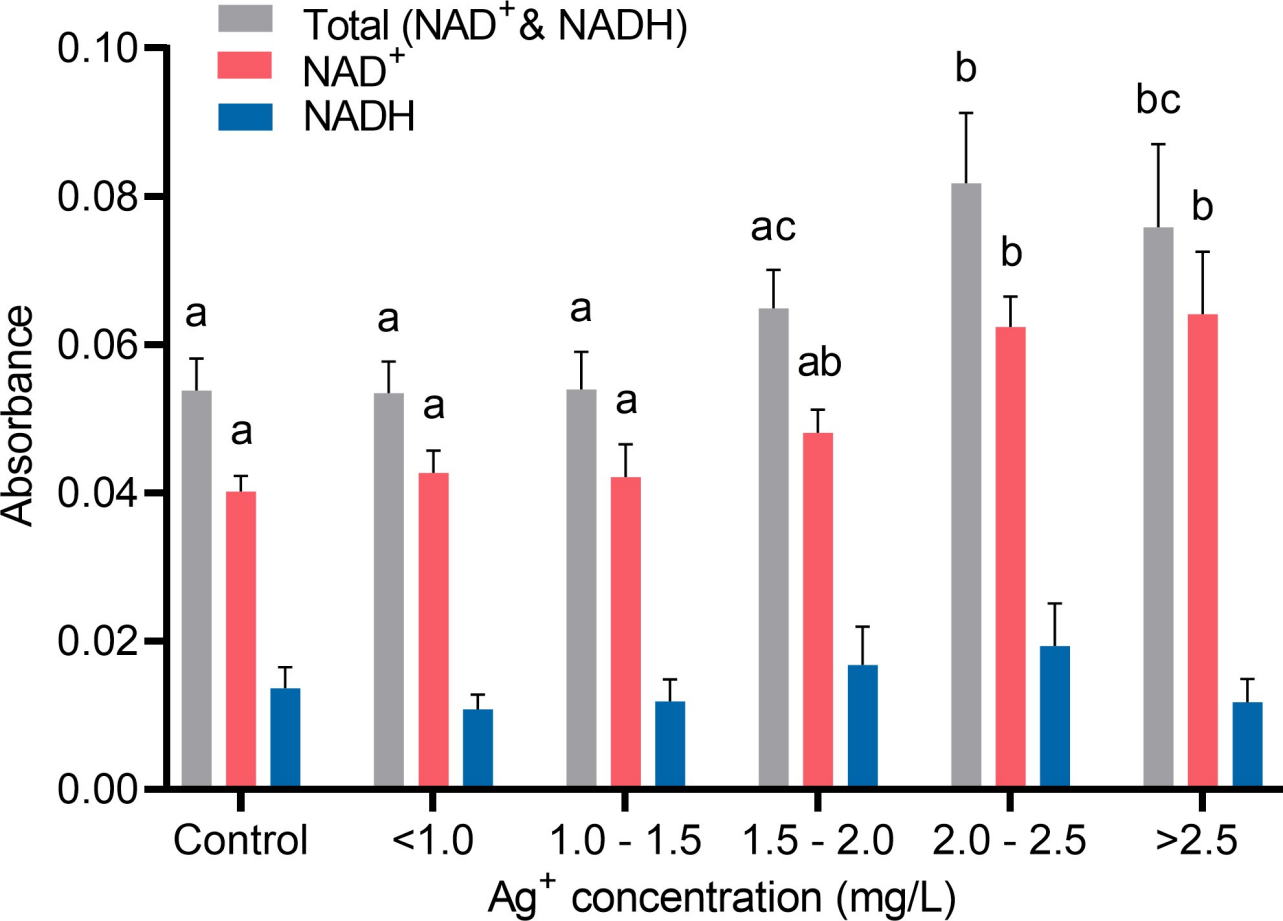
Quantifications of cellular NAD^+^ and NADH from cells in response to different concentrations of silver ions for 21 hours. Cells were grouped by similar ranges of silver ions concentrations treated. Absorbance signals of total NAD^+^ and NADH pool (grey), NAD^+^ (red), and NADH (blue) read at 460 nm were used as indicators of the cellular NAD^+^/NADH concentrations. Values were normalized to living cell number. Means ± SEM. a, b, c = different superscripts denote statistically significant differences in either total NAD^+^ and NADH, NAD^+^, or NADH values among groups (P < 0.05). Experiments were repeated four times.

In order to find out whether the changes of ATP production in cells treated with silver ions were related to oxidation phosphorylation which is the major source of ATP in fibroblasts, NAD^+^ and NADH level in fibroblast cells were analyzed. As shown in Fig 12, when treated with a relatively higher level of silver ions, 2.00 to 2.50 mg/L or > 2.50 mg/L, there was a significant rise of the total pool of NAD^+^ and NADH in cells. However, the level of NADH in all groups of cells treated with different concentrations of silver ions was stable. Consequently, the rise of the total pool of NAD^+^ and NADH in cells was mainly contributed by an increase of NAD^+^ content. This result suggested silver ions can affect oxidative phosphorylation by elevating the level of the total pool of NAD^+^ and NADH, which leads to an increase of ATP production in fibroblasts.

**Fig 13:**
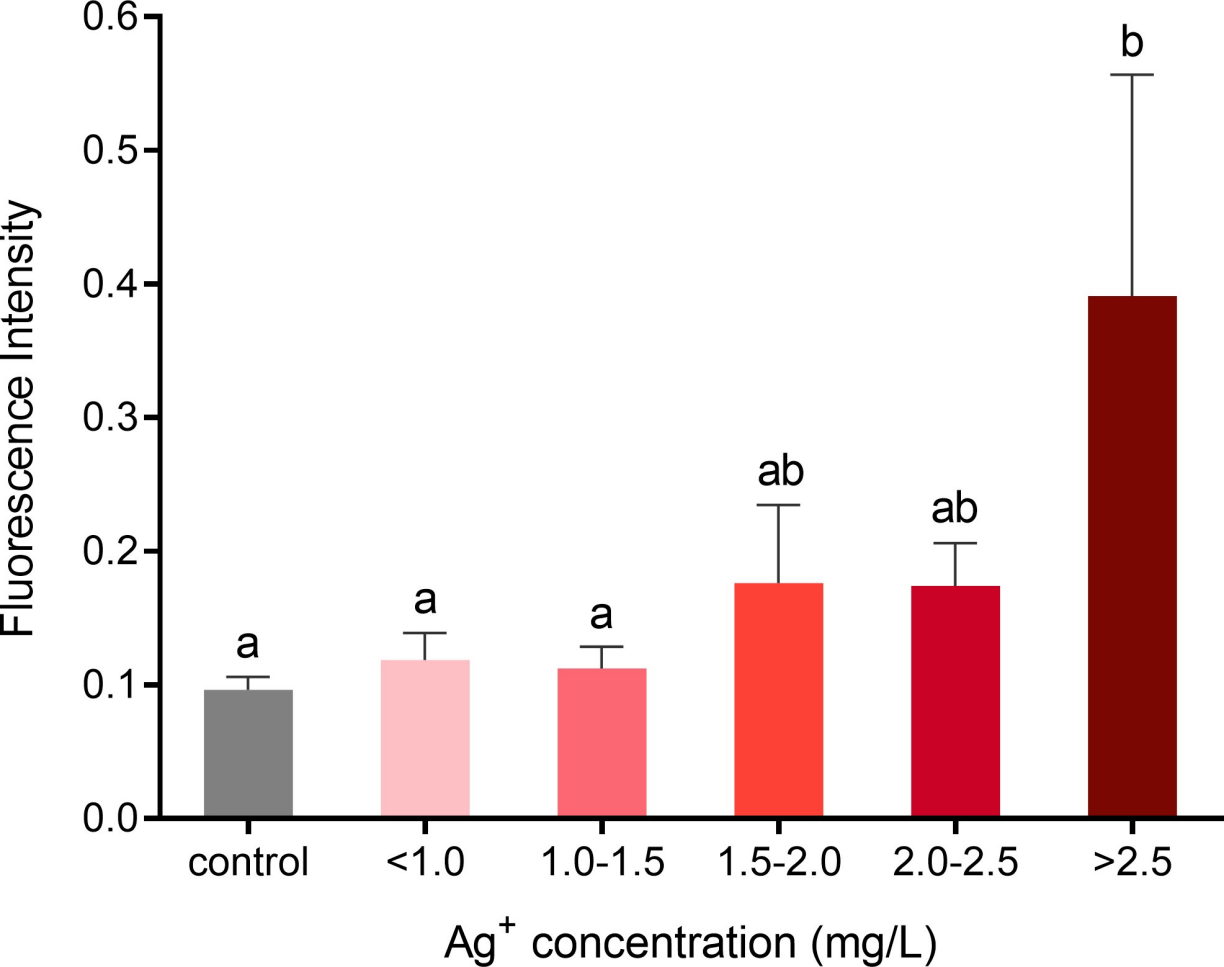
ROS formation in fibroblasts following exposure to different concentrations of silver ions for 21 hours. Cells were grouped by similar ranges of silver ion concentrations treated. Fluorescence signals (Ex/Em = 485/535 nm) were used as indicators of the cellular ROS level. Values were normalized to living cell number. Means ± SEM. a, b, c = different superscripts denote statistically significant differences in ROS values between samples (P < 0.05). Experiments were repeated four times.

The increase of oxidation phosphorylation has been linked to the overproduction of ROS in cells leading to oxidative stress, which results in mitochondrial dysfunction, apoptosis, and cell death. The more NADH that is oxidized, the more ROS will be produced [30]. To investigate whether different concentrations of silver ions increase the production of ROS in porcine fibroblasts, we stained cells with a fluorogenic dye DCFDA to measure hydroxyl, peroxyl, and other ROS activity within the cells. The results show that when cells were exposed to >2.50 mg/L of silver ions, ROS production was elevated significantly compared to the control and lower levels of silver ions (Fig 13). Cells treated with less concentrated silver ions did not show signs of ROS overproduction.

**Fig 14:**
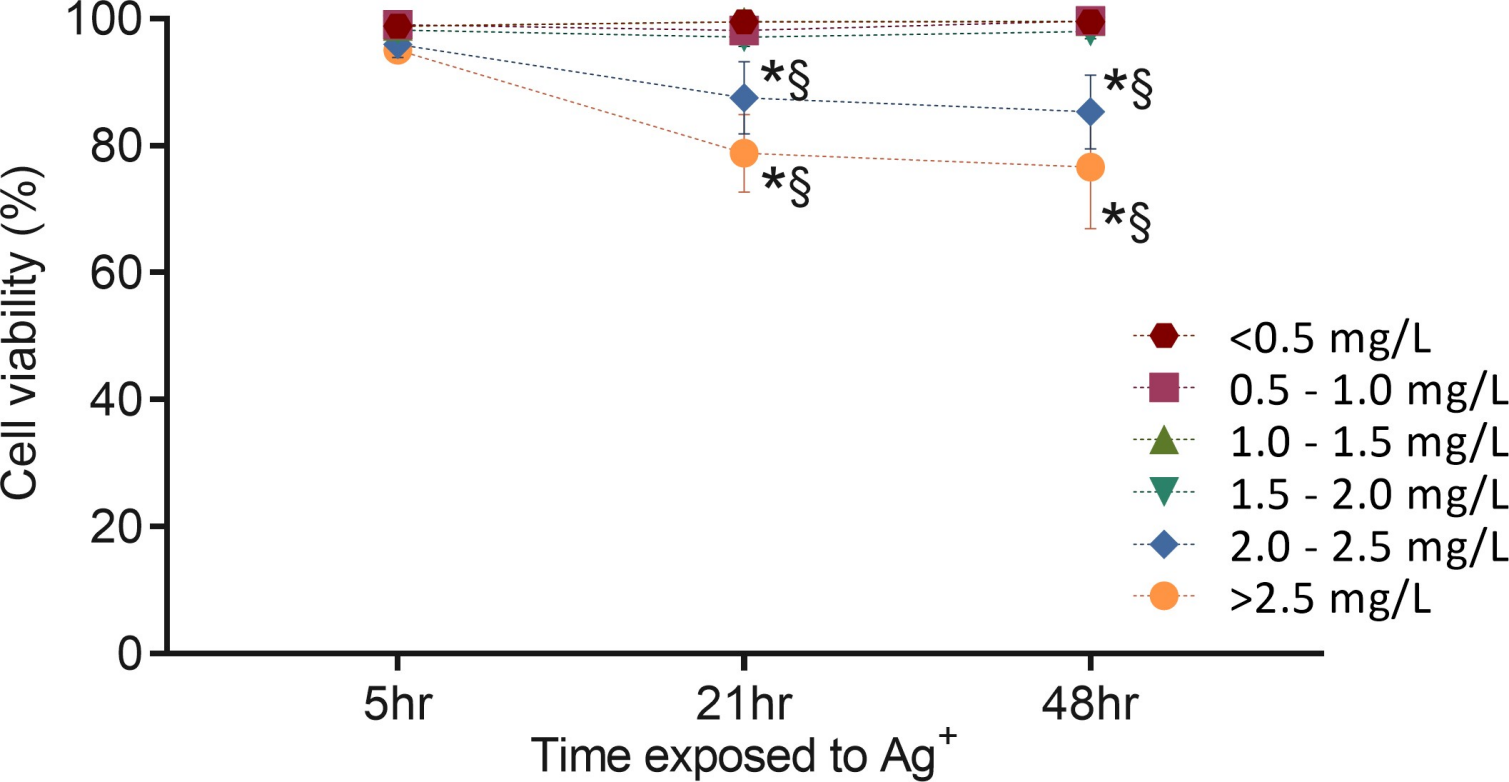
Effects of electrically generated silver ions on the viability of fibroblasts analyzed by flow cytometry. The cells were treated with different concentrations of silver ions for 5, 21, and 48 hours. Cell viability was determined by measuring dead cell number (7-AAD staining) and total cell number (SYTO 13 staining). This figure links to S1 Fig that is one representative of four repeated experiments. Means ± SEM. Cells exposed to different silver ion concentrations (mg/L) were shown in different color symbols: red hexagon (less than 0.50 mg/L including Chamber II and III), red square (0.50 to 1.00 mg/L), green triangle (1.00 to1.50 mg/L), blue inverted triangle (1.50 to 2.00 mg/L), blue rhombus (2.00 to 2.50 mg/L), yellow circle (more than 2.50 mg/L). Two-way ANOVA was performed for statistical analysis (P < 0.05). * = significantly different from the 5-hour exposure with the same silver ion concentration. § = significantly different from < 0.50 mg/L with the same exposure time.

To study the toxicity of silver ions toward fibroblasts, flow cytometry analysis of cell viability was performed following exposure of cells to different concentrations of silver ions for 5, 21, and 48 hours. Cells were labeled with SYTO 13 that stained nucleic acids of all cells and 7-AAD that stained cells with the compromised membrane (S1 Fig). As shown in Fig 14, at a level of less than 2.00 mg/L, silver ions did not affect cell viability despite the time of exposure. In comparison, concentration of silver ions 2.00 to 2.50 mg/L and >2.50 mg/L decreased cell viability to 87.5% and 78.8% respectively, after 21-hour exposure. When exposed for 48 hours, cell viability became slightly lower but remained at a statistically similar level to 21-hour exposure. There was no difference in viability among all groups exposed to silver ion for 5 hours. These results are in line with the data presented in Fig 10 showing the effects of silver ions on cell viability, and demonstrate that the 21-hour exposure treatment could induce a maximized effect of silver ions on cells.

## Discussion

A number of studies have reported changes in cell synthesis, proliferation, metabolism, and migration when cells were exposed to electrical currents of different amplitudes and frequencies. In several wound healing studies, positively or negatively charged cells migrated toward the anode or cathode of an electric field, which is a phenomenon known as galvanotaxis or electrotaxis [31, 32]. Fibroblasts have been reported to migrate either toward the cathode during the proliferative phase of wound healing [33–36] or toward the anode [37]. In the present study, we did not find cell migration at the cathode. This can be explained by the difference in electrical current amplitude, where an absolute lower intensity of current (less than 0.58 nA or 0.35 V) was applied in our study while others delivered a much higher intensity of current (e.g. Yang et al. applied 2.5 mA [36]). The cell morphology changes and cell movements at the anode observed in this study are more likely due to the effects of the release of silver ions from the anode rather than the impact of direct current. Compared with the findings reported by Stoehr et al, where the epithelial cells rounded up and detached from the plastic support after exposed to silver wires and microparticles for 24 hours [38], our fibroblast cells showed similar morphological changes after exposure to electrical biostimulation and silver ions in the present study. In addition, when exposed to silver ions, mesenchymal stem cells displayed cellular shrinkage with less extension/elongation [39]. We also found this type of morphological change in some fibroblast cells when treated with silver ion conditioned culture medium. However, unlike an agglomeration of the wires and the cells Stoehr observed, we found cell aggregation with increased adhesion between the cells and cell clumping. The reason might be the difference in size between materials that were used, where nano-sized silver particles are capable to bind to the surface of the cell wall and membrane [40]. Another possible explanation is that it was silver ion that attracted or bound to the membrane of cells and formed cell clumps. We found that cells moved away from the positive electrode during the electrical biostimulation experiment, which was theoretically the same direction that silver ion was released and flowed to. In cells exposed to silver ion conditioned medium, in which silver ions were homogeneously dispersed, cell clumps were found all over the culture dish. Nevertheless, this idea needs to be further investigated because the direction of spread or the distribution of the released silver ions is not verified in this study, and the mechanism of silver ions interacting with the cell membrane is still largely unknown despite several pieces of evidence from microorganism studies [41, 42]. Overall, the morphology changes in fibroblast cells found in our study are consistent with Becker’s study [9].

Numerous investigations have been conducted to reveal the toxicity effects of nanosilver on different organisms, including laboratory animals [43]. However, it is still unclear whether the toxicity of AgNPs is only due to dissolved Ag^+^ or if only the AgNPs themselves show a toxic effect. An extensive debate is still ongoing regarding this question [20, 39, 44–47]. Regardless, there is no doubt that silver ions at a certain level can affect cellular behavior and induce cytotoxicity. Gao et al [44] exposed mouse embryonic stem cells (mESCs) to the silver ions (silver acetate solution) for 24 hours resulting in nearly complete cell death at the concentration as low as 5.0 mg/L, but interestingly, they found the low concentration of silver ions (up to 0.5 mg/L) increased the rate of cell proliferation. It has been reported that different cell types tend to have different tolerances to silver ions. For example, 1.5 mg/L of silver ions (silver acetate) for T-lymphocytes, 1.0 mg/L for monocytes, and 2.5 mg/L for human mesenchymal stem cells [48]. Moreover, it is important to note that in this study we used electrically generated silver ions that could have different bioavailability than the silver ions produced from silver compounds in other studies where silver salts (e.g. silver nitrate and silver acetate), silver sulfadiazine cream, and silver nanoparticles, are used to determine a mechanism of action of silver. Studies have shown the electrically generated silver ions seemed to be more efficient than silver compounds in antimicrobial activity [14, 22, 49]. In the clinical setting, 5.0 mg/L of silver ions (0.5% silver nitrate) is commonly applied to wounded skin as an antibacterial agent. Nevertheless, lower concentrations of silver ions (0.5 to 5 mg/L, silver acetate) have been shown to be effective at inhibiting bacteria in in vitro studies [48]. Jung et al. even claimed 0.05 to 0.20 mg/L of silver ions significantly eliminated E.coli cells [14]. However, the medium used in the study was PBS which is not commonly used in in vitro studies and cannot resemble applications in the clinic. Indeed, besides the sources of silver ions, chemical compositions of the experimental medium are expected to greatly affect bioavailability due to the interaction of chemicals with dissolved silver ions. Zhang et al found aggregated AgCl limited the bioavailability of free silver ions, thus reduced cytotoxicity of silver ions to mammalian cells [50]. Cell culture and biological media are very complex and vary from study to study. In addition, the fetal bovine serum is commonly used in cell culture media but the level of its contents can be different from batch to batch. However, it is known that the more contents of FBS are in the medium, the lower bioavailability of silver ion has [51]. In this study, the fact that 15% FBS was added to cell culture medium must be taken into account when comparing with other studies.

In response to silver ions, we find morphological changes of fibroblasts were dose-dependent. Lower concentrations of silver ions (0.33 to 2.00 mg/L) did not affect any change to cell morphology, whereas a higher level of silver ions (2.00 to 3.36 mg/L) induced apparent morphological changes in cells, which have also been reported in other studies. Similar to our observation, Cortese-Krott et al. showed low Ag^+^ concentrations (less than 1.72 mg/L) did not affect human fibroblasts morphology, whereas at high concentrations (more than 6.89 mg/L) silver ions affected cell morphology and reduced cell proliferation rate [52]. By using laser scanning microscopy, Herzog et al was able to demonstrate that lung cells changed morphology in form of augmented DNA condensation and alterations in the actin cytoskeleton when exposed to 3.79 mg/L but not for 0.04 and 0.38 mg/L Ag^+^ concentrations [53]. Stoehr et al. found that after exposure to silver nanoparticles and silver ions for 24 hours, human epithelial cells rounded up and detached from the culture dish, most of which were suggested to be dead [38]. In our observation, we indeed find a correlation between morphological change and cell death. However, many live cells with the same morphological change were also observed, indicating that other physiological modifications exist, for example, cell cycle regulation, cytoskeleton organization, and metabolic regulation [54–56]. It has been suggested that cells arrest at the G2/M boundary after treatment with silver nanoparticles [57]. Our results show silver ions reduced cell proliferation so it is possible that the cell cycle was altered in a similar manner as well. However, this speculation needs further confirmation.

In clinical settings, Becker found the exposure of normal human cells in wounds to electrically generated silver ions accelerated healing rates with enhanced effects in many tissues such as bone, soft tissue, nerve, and skins, which suggested either a dedifferentiation process occurring or clonal expansion of pre-existing stem cells [9]. According to our RT-qPCR data, we did not find evidence for silver ion-induced cellular reprogramming or dedifferentiation. The concentration of silver ions in Becker’s clinical studies was not determined, even though the electrical current used to generate silver ion was similar to ours, there may be a difference in the concentrations of silver ions used by Becker and us. The possibility that cells exposed to silver ion were undergoing reprogramming at a very early stage cannot be completely ruled out. After all, the reprogramming of fibroblasts to pluripotency is a stepwise process [58–60] and takes approximately 3–4 weeks [6]. Markers associated with pluripotency including *OCT4*, *SOX2*, *NANOG*, and *KLF4* are not activated until a late stage of reprogramming where downregulation of somatic markers (*THY1* and *collagens*) and reactivation of the embryonic marker stage-specific embryonic antigen 1 (*SSEA1*) have already completed [61]. Therefore, cells exposed to silver ions for such a short period (21 hours) seemed to be not sufficient to remove all the molecular barriers and to activate the pluripotency markers. In order to provide sufficient time for reprogramming, cells should be maintained under an environment that favors undifferentiation of cells. A more suitable stem cell culture technique is needed for future studies.

Furthermore, the RNA-sequencing analysis also shows that no pluripotency genes of the treated cells were affected after exposure to silver ions, but the expression of several genes associated with cellular metabolism was significantly changed. Previous studies [16, 62, 63] suggested that in bacteria, silver ions can interact with the enzymes of the respiratory chain affecting ATP synthesis. The up-regulation of NADH dehydrogenase subunits (*ND1-4*, *ND4L*, *ND5-6*) was observed in the current study. Along with higher expressed ATP synthase subunits (*ATP6* and *ATP8*) and cytochrome oxidase subunits (*COX1*, *COX2*, *COX3*, and *CYTB*), our data suggested an increase of oxidative phosphorylation was induced in cells in response to silver ions exposure. This increase of oxidative phosphorylation can potentially lead to oxidative stress when the reactive oxygen species (ROS), a byproduct of oxidative phosphorylation, are overproduced. Numerous findings were reported previously that the exposure of silver ions can induce oxidative stress through the generation of ROS, some examples can be reviewed here [39, 45, 46, 52, 64]. Since most of the cells exposed to a relatively low concentration of silver ions in the current study showed no adverse effects on viability, it is possible that there was an increase in oxidative phosphorylation without generating excess ROS (as shown in Fig 13). In order to maximize the effects of silver ion exposed to cells or bacteria, most research or clinical applications have chosen 24 hours of incubation time that allows silver ions to be taken up by the cells, to interact with the intracellular compartments, and to develop any effects fully [57]. In this study, we also evaluated the effect of silver ions on cell viability by flow cytometry at different time points, 5, 21, and 48 hours. In terms of cell viability, 5 hour-exposure to silver ions at all concentrations tested here was too early to show any effects. This effect on viability kept developing to 21 hours of exposure and maintained until 48 hours of exposure without significant intensification. However, any early or late cellular responses besides cell apoptosis cannot be completely excluded and further investigations are needed.

In one study, intracellular ATP depletion of human fibroblasts was found as early as 8-hour of exposure to silver nitrate, as well as 24-hour exposure [51]. However, in the present study, elevated production of intracellular ATP was observed when cells were treated with a lower concentration of silver ions (1.50 to 2.00 mg/L). ATP production dropped to a normal level (similar to the control) with higher concentrations of silver ions (>2.50 mg/L) exposure. The discrepancy of the results in that study with ours is that the number of dead cells was excluded from the calculation of ATP content here, while the whole population of cells (live and dead) was taken into account in that study. A decrease of ATP content could be the result of dead cells, which do not contain intracellular ATP, as well as reduced intracellular ATP in sub-lethally damaged cells [51]. By excluding dead cells from the determination of the ATP content level, we are able to detect the intracellular ATP in live cells. Our results suggest that ATP production was elevated in cells that remained alive and integral after treated with a relatively lower level of silver ions (1.50 to 2.00 mg/L) for 21 hours.

The precise mechanism of antimicrobial actions and metabolic regulations are yet to be clarified. Previous studies proposed that the reduced form of nicotinamide adenine dinucleotide (NADH) and NADH dehydrogenases (Complex I) play a significant role in the reduction of silver ion to metallic silver, where the electron from NADH is donated to silver ion [65–68]. Usually, NADH is oxidized by giving its electrons to Complex I of the electron transport chain (ETC) and sequentially passing to the downstream of ETC, resulting in the reduction of oxygen to water eventually. In the present study, an increased level of the NADH and NAD^+^ pool was found in cells treated with 2.00 to 2.50 mg/L of silver ions (Fig 12), which was mainly contributed by the increase of NAD^+^ content but not the NADH. Our results suggest that there was increased oxidative phosphorylation activity, and more NADH molecules had lost their electrons that were probably accepted by silver ions. This is in accordance with the RNA-seq result (Table 1), where up-regulation of NADH dehydrogenase subunit genes was found. Moreover, Complex I is a major source of reactive oxygen species (ROS) production in mammalian mitochondria. ROS play a pivotal role in various physiological and pathological processes acting as secondary messenger-signaling molecules [69]. Overproduction of ROS has been known to cause oxidative stress to the cells that can eventually induce apoptosis and cell death. Silver has been reported to evoke the intracellular production and extracellular release of ROS [52, 57, 70]. However, the mechanism of how silver ion induces ROS production is still unknown. These results are in line with ours that a high concentration of electrically generated silver ions increased intracellular ROS level (Fig 13). When treated with lower levels of silver ions, ROS production was not higher in the cells that did not cause any oxidative stress. This explains the reason why cell viability did not drop significantly under these concentrations of silver ions (Fig 10). It is known that somatic cells mainly generate energy through oxidative phosphorylation and pluripotent cells rely on glycolytic metabolism. During cell reprogramming, metabolic remodeling from oxidative phosphorylation to glycolytic metabolism is essential as it mediates the process, and any perturbations to this shift can affect reprogramming efficiency [71–77]. Recently, it has been reported that there is a transient burst of oxidative phosphorylation activity with increased ATP, NADH, and ROS at the very early stage of the reprogramming of fibroblasts into pluripotent cells [78, 79]. Interestingly, the fibroblasts treated with approximately 2.00 mg/L of electrically generated silver ions in our experiments exhibited cellular biochemical characteristics that are similar to the burst of oxidative phosphorylation seen during early cell reprogramming [80]. Our results suggest that the potential of silver ions inducing an oxidative phosphorylation burst is possible and could provide a supplemental explanation to the studies that showed silver ions can promote tissue regeneration and wound healing while preventing bacterial infection [9, 81–83].

## Conclusions

In this study, we attempted to unravel the effects of morphological and physiological changes of porcine fibroblast cells exposed to electrical biostimulation. It was mainly the electrically generated silver ion that was responsible for the cellular changes observed in the present work, including morphological changes and gene expression changes. Silver ion exposure for 21 hours did not increase the expression of pluripotency genes and did not reprogram fibroblast cells to an undifferentiated state as we hypothesized. However, upon characterizing transcriptomic changes using RNA-sequencing, the results suggested most functions and pathways impacted are associated with various processes of cellular metabolism, which indicated an increase of oxidation phosphorylation in the silver ions treated cells. Additionally, the investigations towards the change of key metabolites further confirmed that low concentrations of silver ions could boost oxidative phosphorylation without causing cell death. It is known that silver ion is an antibacterial agent that can inhibit the proliferation of bacteria during wound healing. The findings in this study demonstrated additional effects of silver ions on metabolic function alterations. The valuable benefits of silver in health care have been recognized for thousands of years but the concern of adverse and toxic effects towards mammalian cells should not be ignored as well. As the application of silver products is rapidly growing in a variety of fields [84], it is necessary for future studies to evaluate and determine the optimal form and concentration of silver that will be utilized. The electrically generated silver ion as a novel method for treating diseases, as compared to silver nanoparticles and silver salts, should be investigated further in the studies of animal and human healthcare, as well as the clinical applications of silver.

## Supporting information

S1 Table Primers used with the Fluidigm BioMark qPCR platform. (DOCX)

S2 Table List of genes for RNA-sequencing data found to be significantly differentially expressed in cells exposed to silver ions and control. (DOCX)

S1 Fig Representative dot-plot profiles of fibroblast cells in response to silver ions. 3.29 mg/L of silver ions were generated from electrical current and a series of 2:10 dilution (2.63 mg/L, 1.97 mg/L, 1.31 mg/L, 0.66 mg/L) was prepared to treat cells for 5, 21, and 48 hours. Chamber II and Chamber III were included as controls. Cells were labeled with 7-AAD and SYTO 13 for flow cytometry analysis. Damaged or dead cells (UR—red) were discriminated from live cells (LR—green) by the quadrants. Debris was gated out using a forward scatter (FSC) VS side scatter (SSC) plot. Experiments were repeated four times.
(TIF)
