# Supplementary material for: Effects of electrical biostimulation and silver ions on porcine fibroblast cells
Source: PLoS One. 2021 Feb 10;16(2):e0246847. doi: 10.1371/journal.pone.0246847 (PMC7875371; doi:10.1371/journal.pone.0246847)
Supplement: S1 Table — (DOCX) [file pone.0246847.s001.docx]

**S1 Table. Primers used with the Fluidigm BioMark qPCR platform.**

| Functions | Gene  Symbol | 5' - Forward Primer - 3' | 5' - Reverse Primer - 3' |
| --- | --- | --- | --- |
| Apoptosis | **ATM** | TTAAGAGCTTGGGCTCTGGAA | TGGTACAGCGAGATCACACA |
|  | **BCL2L1** | AGCGTAGACAAGGAGATGCA | TTCAGGTAAGTGGCCATCCA |
|  | **CASP3** | TCAGAGGGGACTGCTGTAGAA | CGTCTCAATCCCACAGTCCAA |
|  | **TP53** | TCGCCAGTGCAAAAGAAGAA | GGAACATCTCGAAGCGTTCA |
|  | **XIAP** | GAGTGCTCAGAAAGACAATGCA | CCTCAGCTGTTCTTCAGCACTA |
| Epigenetic modifiers | **ASH2L** | TTTCCGGCCATCTCACTGTA | TCCTTCGGCGGATACTTGAA |
|  | **DMAP1** | GACGGAGCCATGTTCTTCCA | ACCTGCACCGTCTTGTTGAA |
|  | **DNMT1** | AAAGGCGCTCATAGGCTTCA | ACGCTGAACAGTGGTGCATA |
|  | **DNMT3A** | ATGACCTCTCCATCGTCAACC | CAGGAGGCGGTAGAACTCAAA |
|  | **DNMT3B** | AGCTGTACCCTGCCATTCC | AAGTACCCTGTTGCGATTCCA |
|  | **EHMT2** | ACTTCAGCCTCTACTACGATTCC | GTTCAGCCAGAGCTTCAACC |
|  | **EZH2** | CGAAGGATCCAGCCTGTTCA | ACGGGATGACTTGTGTTGGAA |
|  | **HDAC3** | ATCGATTGGGCTGCTTCAAC | GAGGGATGTTGAAGCTCTTGAC |
|  | **SIRT1** | TGTCAGAGTTACCACCCACAC | ACTGAAGAAGCTGGTGGTGAA |
| House-keeping | **EIF4A1** | GAGAAGCCCTCTGCCATCC | ATTGGGCTTGAGCGATCACA |
|  | **GAPDH** | AGTGGACATTGTCGCCATCA | CGTGGGTGGAATCATACTGGAA |
|  | **HPRT1** | AAAGAGATGGGAGGCCATCA | GTAATCCAGCAGGTCAGCAAA |
|  | **HSP90AA1** | GACCAGAAACCCCGATGACA | GATCCTCCCAGTCATTGGTCAA |
|  | **RPN1** | AGACAGTGGGATCTCCTCCA | GGTGGAAACATTGCCAATCTCA |
|  | **TAF11** | AGAGAAGAAGCAGAAAGTGGATGAA | GGTTCAGCTGCTCCTCAGAA |
| Imprinted | **GNAS** | AAGGCAGAGGAGAAGAAGCA | GATGGGTCCCCTCTTGGAA |
|  | **GRB10** | AAGCACGCGGATGAATATCCTA | AGTGCTGCGTCCTGTGAA |
|  | **IGF2** | CAGCCCACAGCGATTCCAA | GAGGCCAAGGCCAAGAAGAC |
|  | **IGF2R** | TGTGGTGGTGGCAAGAGAATA | CAGCACTGGAGCACTCTCTAA |
|  | **NDN** | ATGTGGTACGTGCTGGTCAA | CACTTCTTGTAACTGCCGATGAC |
|  | **NNAT** | TTTCGAAATCCTCCAGGGACAC | CCAGCTTCTGCAGGGAGTAC |
|  | **PEG10** | AGTCCTCGCGTGGTGAGTA | CCCAGGTGTAGCTTCACTCC |
|  | **UBE3A** | ACTTTTCGTGACTTGGGAGAC | CTTCCACACTTCCTTCATACTCC |
| Maternal effect | **BMP15** | GGCCATTGGTTAATGGAGCAA | GCTACCCGGTTTGGTCTCA |
|  | **GDF9** | AACACTGTCCGGCTCTTCAC | TCAACAGCAGTAACACGATCCA |
|  | **MOS** | GGCTTCGGCTCGGTATACAA | GTTCTTGGTGCATCTGCTCAC |
|  | **NOBOX** | CCACTATCCGGACAGCGAAA | CCGGCGATTCTGGAACCA |
|  | **ZAR1** | CCCTTATCGCGTGGAGGATA | TCCACGTGGCGAAGTTTTAC |
|  | **ZP3** | CACCGTAATGGTGGAGTGTCA | CCTGATGAGCTTCCCGGTAC |
| Pluripo-tency | **KLF4** | GGGAAGGGAGAAGACACTG | TCTTTGCTTCATGTGGGAGA |
|  | **LIN28A** | TTCGGCTTCCTGTCCATGAC | GCCCTCCATGTGCAGCTTA |
|  | **MYC** | CGAACCCTTGGCTCTCCA | GCTGCCTCTTTTCCACAGAAA |
|  | **NANOG** | CTTGGAAACTGCTGGGGAAA | CCATGATTTGCTGCTGGGTA |
|  | **POU5F1** | AGAAGAGGATCACCCTGGGATA | ATGGTCGTTTGGCTGAACAC |
|  | **SOX2** | CCTGCAGTACAACTCCATGAC | TGCGAGTAGGACATGCTGTA |
| Sexing | **SRY** | GCTCAAACGATGGACGTGAAA | CGTTCATGGGTCGCTTGAC |
| Tropho-blast  related | **ASCL2** | CTGGTGAACTTGGGGTTCCA | GAGCGCAGCGTCTCCA |
|  | **CYP17A1** | GGACACAGATGTCGTCGTCAA | AAGCGCTCAGGCATGAACA |
|  | **ELF5** | AGTGGCATCAAAAGCCAAGAC | GCAGGTCTCGTACAAATTCCC |
|  | **HAND1** | GCGAGAGCAAGCGGAAAA | CCTGTGCGCCCTTTAATCC |
|  | **HSD17B1** | TCGGGTCGCATATTGGTGAC | AAACTTGCTGGCGCAGTAAAC |
|  | **KRT8** | AAGCGTACCGACATGGAGAA | TCCAGCTCGACCTTGTTCA |
|  | **TEAD4** | TGTTGGAGTTCTCTGCCTTCC | GGCCGATGTGCACAAACAA |
