# Supplementary material for: Effects of electrical biostimulation and silver ions on porcine fibroblast cells
Source: PLoS One. 2021 Feb 10;16(2):e0246847. doi: 10.1371/journal.pone.0246847 (PMC7875371; doi:10.1371/journal.pone.0246847)
Supplement: S2 Table — (DOCX) [file pone.0246847.s002.docx]

**S2 Table. List of genes for RNA-sequencing data found to be significantly differentially expressed in cells exposed to silver ions and control.**

| Gene ID | Gene symbol | trt. mean. count | ctl. mean. count | Log_2_FC | rawP | adjP |
| --- | --- | --- | --- | --- | --- | --- |
| ENSSSCG00000007197 | RSPO4 | 22.67 | 0.00 | 23.09 | 4.55E-18 | 0.00E+00 |
| ENSSSCG00000021405 |  | 18.33 | 0.00 | 23.09 | 5.77E-18 | 0.00E+00 |
| ENSSSCG00000012637 |  | 20.67 | 0.00 | 22.94 | 1.04E-17 | 0.00E+00 |
| ENSSSCG00000014079 |  | 16.00 | 0.00 | 22.90 | 1.77E-17 | 0.00E+00 |
| ENSSSCG00000030300 | MT2A | 2846.67 | 15.00 | 10.34 | 3.19E-15 | 0.00E+00 |
| ENSSSCG00000007045 | CHGB | 247.00 | 0.00 | 13.37 | 3.94E-11 | 0.00E+00 |
| ENSSSCG00000009979 |  | 253.00 | 0.33 | 12.32 | 1.50E-10 | 0.00E+00 |
| ENSSSCG00000003921 |  | 134.67 | 0.00 | 12.56 | 4.54E-10 | 0.00E+00 |
| ENSSSCG00000013382 | PLEKHA7 | 26.33 | 0.00 | 23.50 | 1.84E-09 | 0.00E+00 |
| ENSSSCG00000012855 |  | 53.67 | 0.00 | 11.17 | 2.74E-08 | 0.00E+00 |
| ENSSSCG00000028804 | CCDC181 | 87.67 | 0.00 | 11.71 | 5.07E-08 | 1.00E-04 |
| ENSSSCG00000030368 | HSP70.2 | 27540.00 | 1873.00 | 6.62 | 6.96E-08 | 1.00E-04 |
| ENSSSCG00000007554 | ZFAND2A | 1898.67 | 131.33 | 6.45 | 1.65E-07 | 2.00E-04 |
| ENSSSCG00000029333 | RASD1 | 428.33 | 33.67 | 6.45 | 2.98E-07 | 3.00E-04 |
| ENSSSCG00000014986 | MMP3 | 58.00 | 0.00 | 10.31 | 5.83E-07 | 5.00E-04 |
| ENSSSCG00000015595 | ATF3 | 719.00 | 70.67 | 6.14 | 1.06E-06 | 8.00E-04 |
| ENSSSCG00000018063 |  | 1227339.33 | 87301.00 | 6.76 | 1.95E-06 | 1.40E-03 |
| ENSSSCG00000025317 |  | 31.00 | 0.00 | 10.58 | 2.26E-06 | 1.60E-03 |
| ENSSSCG00000028335 | U3 | 73.00 | 0.67 | 10.04 | 3.62E-06 | 2.30E-03 |
| ENSSSCG00000000442 | INHBE | 0.33 | 315.33 | -7.94 | 4.43E-06 | 2.70E-03 |
| ENSSSCG00000018700 | RNase_MRP | 21.67 | 0.00 | 9.40 | 5.25E-06 | 3.00E-03 |
| ENSSSCG00000009996 | LIF | 77.00 | 5.67 | 6.86 | 5.28E-06 | 3.00E-03 |
| ENSSSCG00000009585 | GADD45G | 776.33 | 47.67 | 7.01 | 1.30E-05 | 6.70E-03 |
| ENSSSCG00000015025 | CRYAB | 2765.67 | 403.00 | 5.53 | 1.31E-05 | 6.70E-03 |
| ENSSSCG00000021569 | MMP25 | 579.33 | 45.00 | 6.62 | 1.63E-05 | 8.00E-03 |
| ENSSSCG00000010537 | GOT1 | 2143.33 | 290.33 | 5.69 | 1.68E-05 | 8.00E-03 |
| ENSSSCG00000018084 | ND3 | 21605.67 | 2805.67 | 5.90 | 1.94E-05 | 8.90E-03 |
| ENSSSCG00000002274 | HSPA2 | 807.67 | 142.00 | 5.12 | 2.87E-05 | 1.26E-02 |
| ENSSSCG00000007563 | BRAT1 | 388.00 | 68.00 | 5.48 | 3.12E-05 | 1.32E-02 |
| ENSSSCG00000018076 |  | 29191.00 | 3793.33 | 5.90 | 3.24E-05 | 1.32E-02 |
| ENSSSCG00000002960 | RASGRP4 | 248.33 | 9.00 | 7.62 | 3.31E-05 | 1.32E-02 |
| ENSSSCG00000013008 | CDCA5 | 853.67 | 166.00 | 5.11 | 3.49E-05 | 1.35E-02 |
| ENSSSCG00000005645 | TRUB1 | 531.33 | 58.33 | 6.04 | 3.78E-05 | 1.40E-02 |
| ENSSSCG00000001759 | DNAJA4 | 286.00 | 22.67 | 6.12 | 3.85E-05 | 1.40E-02 |
| ENSSSCG00000007603 | NPTX2 | 260.00 | 23.00 | 6.32 | 4.53E-05 | 1.60E-02 |
| ENSSSCG00000024853 | ssc-mir-4332 | 1777.00 | 322.33 | 5.27 | 5.98E-05 | 2.05E-02 |
| ENSSSCG00000018086 | ND4L | 8823.33 | 1638.33 | 5.41 | 6.17E-05 | 2.05E-02 |
| ENSSSCG00000014349 | TMEM173 | 23.67 | 0.00 | 9.45 | 6.36E-05 | 2.06E-02 |
| ENSSSCG00000002297 | RDH12 | 32.00 | 2.33 | 6.69 | 7.13E-05 | 2.25E-02 |
| ENSSSCG00000000181 | RND1 | 209.33 | 8.67 | 7.58 | 7.67E-05 | 2.36E-02 |
| ENSSSCG00000016600 | TMEM229A | 122.00 | 7.33 | 6.99 | 8.62E-05 | 2.53E-02 |
| ENSSSCG00000001977 | STXBP6 | 0.00 | 148.00 | -7.45 | 8.76E-05 | 2.53E-02 |
| ENSSSCG00000004858 | SALL3 | 18.00 | 0.00 | 9.83 | 8.82E-05 | 2.53E-02 |
| ENSSSCG00000018075 | COX1 | 583338.67 | 108814.00 | 5.38 | 9.19E-05 | 2.53E-02 |
| ENSSSCG00000018087 | ND4 | 183665.67 | 34792.33 | 5.37 | 9.48E-05 | 2.53E-02 |
| ENSSSCG00000009648 | NEFM | 9833.00 | 2138.33 | 5.16 | 9.85E-05 | 2.53E-02 |
| ENSSSCG00000018094 | CYTB | 110286.67 | 21717.00 | 5.30 | 1.01E-04 | 2.53E-02 |
| ENSSSCG00000020149 | 5_8S_rRNA | 2205.67 | 508.33 | 4.90 | 1.03E-04 | 2.53E-02 |
| ENSSSCG00000018069 | ND2 | 52419.67 | 10185.00 | 5.32 | 1.04E-04 | 2.53E-02 |
| ENSSSCG00000011763 | MRPL47 | 306.67 | 60.00 | 5.23 | 1.05E-04 | 2.53E-02 |
| ENSSSCG00000023048 | Metazoa_SRP | 1113.33 | 248.67 | 5.13 | 1.05E-04 | 2.53E-02 |
| ENSSSCG00000007949 |  | 24.00 | 0.33 | 9.29 | 1.07E-04 | 2.53E-02 |
| ENSSSCG00000018078 | COX2 | 184574.33 | 36115.67 | 5.30 | 1.09E-04 | 2.54E-02 |
| ENSSSCG00000018065 | ND1 | 75446.67 | 15256.67 | 5.27 | 1.24E-04 | 2.83E-02 |
| ENSSSCG00000018092 | ND6 | 35612.33 | 7320.00 | 5.27 | 1.29E-04 | 2.86E-02 |
| ENSSSCG00000018080 | ATP8 | 15996.67 | 3143.67 | 5.37 | 1.32E-04 | 2.86E-02 |
| ENSSSCG00000018091 | ND5 | 107969.67 | 23015.67 | 5.19 | 1.32E-04 | 2.86E-02 |
| ENSSSCG00000018082 | COX3 | 236418.67 | 49107.67 | 5.22 | 1.42E-04 | 2.97E-02 |
| ENSSSCG00000018061 |  | 40337.33 | 8343.67 | 5.21 | 1.42E-04 | 2.97E-02 |
| ENSSSCG00000011113 |  | 90.67 | 7.67 | 6.19 | 1.56E-04 | 3.20E-02 |
| ENSSSCG00000015235 | ETS1 | 504.33 | 123.67 | 4.93 | 1.66E-04 | 3.36E-02 |
| ENSSSCG00000003563 | GPN2 | 347.67 | 56.33 | 5.05 | 1.74E-04 | 3.47E-02 |
| ENSSSCG00000018081 | ATP6 | 205697.00 | 44388.67 | 5.16 | 1.81E-04 | 3.53E-02 |
| ENSSSCG00000014032 | NHP2 | 2.33 | 297.67 | -6.20 | 1.90E-04 | 3.67E-02 |
| ENSSSCG00000017555 | LUC7L3 | 4358.00 | 1105.67 | 4.86 | 2.03E-04 | 3.85E-02 |
| ENSSSCG00000008963 | AREG | 1154.67 | 292.67 | 4.69 | 2.15E-04 | 4.02E-02 |
| ENSSSCG00000005178 | CNTLN | 0.33 | 138.00 | -6.68 | 2.27E-04 | 4.18E-02 |
| **trt**: treatment group (silver ions treated cells). **ctl**: control group (normal porcine fibroblast cells). **Log_2_ FC**: Log_2_ fold change. **rawP**: raw P-values. **adjP**: adjusted P-values (Benjamini and Hochberg’s) | | | | | | |
