# Supplementary figures and images for: Effects of electrical biostimulation and silver ions on porcine fibroblast cells
Source: PLoS One. 2021 Feb 10;16(2):e0246847. doi: 10.1371/journal.pone.0246847 (PMC7875371; doi:10.1371/journal.pone.0246847)

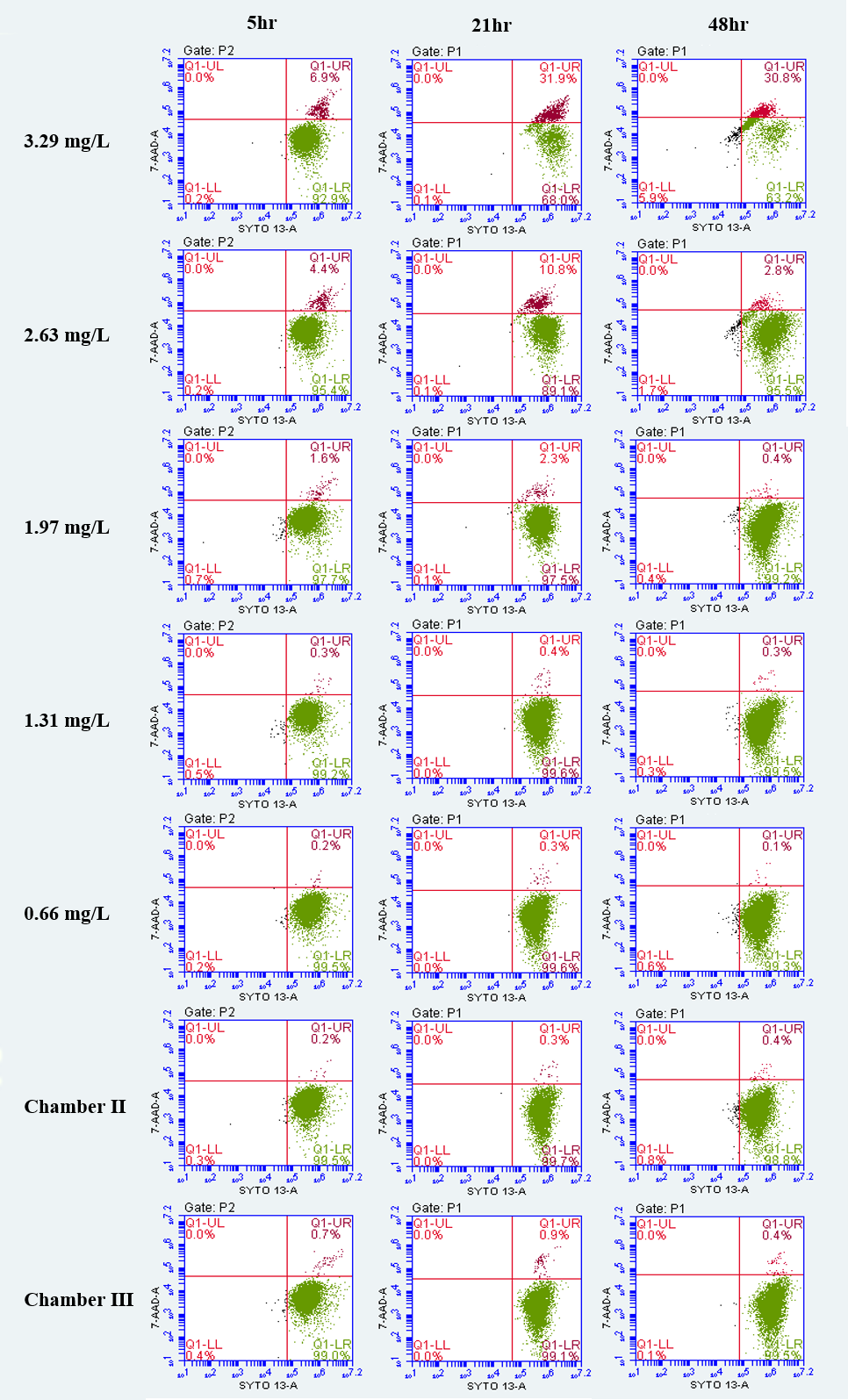

Supplement: S1 Fig — 3.29 mg/L of silver ions were generated from electrical current and a series of 2:10 dilution (2.63 mg/L, 1.97 mg/L, 1.31 mg/L, 0.66 mg/L) was prepared to treat cells for 5, 21, and 48 hours. Chamber II and Chamber III were included as controls. Cells were labeled with 7-AAD and SYTO 13 for flow cytometry analysis. Damaged or dead cells (UR—red) were discriminated from live cells (LR—green) by the quadrants. Debris was gated out using a forward scatter (FSC) VS side scatter (SSC) plot. Experiments were repeated four times. (TIF) [file pone.0246847.s003.tif]
